# Traceable stimulus-dependent rapid molecular changes in dendritic spines in the brain

**DOI:** 10.1038/s41598-020-72248-4

**Published:** 2020-09-17

**Authors:** Kazuya Kuboyama, Takafumi Inoue, Yuki Hashimotodani, Takuya Itoh, Tohsuke Suzuki, Aya Tetsuzawa, Yosuke Ohtsuka, Ryo Kinoshita, Ren Takara, Tohru Miyazawa, Pooja Gusain, Masanobu Kano, Maki K. Yamada

**Affiliations:** 1 Department of Neuropharmacology, Kagawa School of Pharmaceutical Sciences and Institute of Neuroscience, Tokushima Bunri University, 1314-1 Shido, Sanuki, Kagawa 769-2193 Japan; 2 Department of Life Science and Medical Bioscience, School of Advanced Science and Engineering, Waseda University, 2-2 Wakamatsu-cho, Shinjuku, Tokyo 162-8480 Japan; 3 Department of Neurophysiology, Graduate School of Medicine, The University of Tokyo, 7-3-1 Hongo, Bunkyo-ku, Tokyo 113-0033 Japan; 4 Laboratory for Neural Circuit Systems, Institute of Neuroscience, Tokushima Bunri University, 1314-1 Shido, Sanuki, Kagawa 769-2193 Japan; 5 Department of Neurophysiology, Graduate School of Medicine and International Research Center for Neurointelligence, The University of Tokyo Institutes for Advanced Study, The University of Tokyo, 7-3-1 Hongo, Bunkyo-ku, Tokyo 113-0033 Japan; 6 Present Address: Department of Developmental and Regenerative Neurobiology, Institute of Brain Science, Nagoya City University Graduate School of Medical Sciences, 1, Kawasumi, Mizuho-cho, Mizuho-ku, Nagoya, Aichi 467-8601 Japan

**Keywords:** Cellular neuroscience, Neural circuits, Molecular neuroscience, Learning and memory

## Abstract

Dendritic spines function as microcompartments that can modify the efficiency of their associated synapses. Here, we analyzed stimulus-dependent molecular changes in spines. The F-actin capping protein CapZ accumulates in parts of dendritic spines within regions where long-term potentiation has been induced. We produced a transgenic mouse line, AiCE-Tg, in which CapZ tagged with enhanced green fluorescence protein (EGFP-CapZ) is expressed. Twenty minutes after unilateral visual or somatosensory stimulation in AiCE-Tg mice, relative EGFP-CapZ signal intensification was seen in a subset of dendritic spines selectively in stimulated-side cortices; this right-left difference was abolished by NMDA receptor blockade. Immunolabeling of α-actinin, a PSD-95 binding protein that can recruit AMPA receptors, showed that the α-actinin signals colocalized more frequently in spines with the brightest EGFP-CapZ signals (top 100) than in spines with more typical EGFP-CapZ signal strength (top 1,000). This stimulus-dependent in vivo redistribution of EGFP-CapZ represents a novel molecular event with plasticity-like characteristics, and bright EGFP-CapZ in AiCE-Tg mice make high-CapZ spines traceable in vivo and ex vivo. This mouse line has the potential to be used to reveal sequential molecular events, including synaptic tagging, and to relate multiple types of plasticity in these spines, extending knowledge related to memory mechanisms.

## Introduction

Dendritic spines are small protrusions that work as microcompartments and provide structural delimitation for glutamatergic postsynaptic sites. Spine morhphology is highly variable, ranging in shape from thin filopodia to large mushroom-like heads. Accumulating evidence has indicated that structural plasticity of dendritic spines is an important mediator of neuroplasticity^1–3^. Meanwhile, structural remodeling in particular spines has been related to long-term potentiation (LTP) of synaptic currents^4,5^ in those spines in vitro^6^. However, an immediate enlargement of spines in vivo has not been clearly demonstrated, perhaps, at least partially due to their sparsity^7^. In addition, the time course of spine remodeling in vivo might be different from that observed in vitro. Notwithstanding, any local changes, including changes in currents and structure, should be preceded by immediate, stimulus-dependent molecular changes that might also serve as a kind of tag for the changes underlying plasticity for memory.

CapZ is a filamentous actin (F-actin) capping protein that exhibits enhanced accumulation in LTP-induced layers^8^. Conversely, low CapZ levels have been found in humans with conditions characterized by memory impairment, including Down syndrome^9^ and Alzheimer disease^10,11^. Upon molecular interaction with CapZ, F-actin, the major cytoskeletal element found in dendritic spines^12^, exhibits an enhanced tendency to form cloud-like^13^ or fluid-like^14^ structures rather than bundled filaments. Meanwhile CapZ is detectable in about one-fourth of spines at varying levels that appear to be independent of F-actin levels or spine size^8^. Although the mechanisms underlying this heterogenous localization of CapZ among spines is unknown, it has been suggested that the association of F-actin with CapZ versus other capping proteins may be involved^15,16^. Additionally, regulated degradation of CapZ might limit its distribution^17^. Our research group became interested in CapZ owing to its emergence as one of seven proteins (among over 400 protein spots in a two-dimensional difference gel electrophoresis assay) whose expression was decreased following memory-impairing fornix lesioning^8,18,19^.

We produced a stable transgenic mouse line, called AiCE-Tg, that expresses an enhanced green fluorescent protein (EGFP)-tagged CapZ (EGFP-CapZ) marker. The EGFP-CapZ fusion protein includes the β2 splice variant of CapZ, which is expressed in the brain^20^, and the native C-terminus region that binds F-actin^21^ and other major binding proteins^22^. Because we have found that stimulus-dependent increases in large spines occur preferentially in Arc-positive neurons^7^, we put this EGFP-CapZ marker under the control of an *Arc*-promoter^23,24^ deprived of any known mRNA-localization signals. The AiCE-Tg mouse line enables direct visualization of CapZ expression by EGFP, even in fixed brain sections, while overcoming the unevenness produced by depth-dependent intensity alterations in immunolabeling methods.

We hypothesized that CapZ expression in spines may be a landmark for ensuing plastic changes. Thus, the aim of this study was to examine whether stimulus-dependent changes in EGFP-CapZ signals in a subset of spines, such as those due to rapid EGFP-CapZ accumulation and/or regulated degradation, could be demonstrated reliably in AiCE-Tg mice. If so, this change, reflecting reorganization of a spine protein upon stimulation, would represent a kind of molecular event consistent with a plasticity phenomenon in spines in vivo.

## Results

### EGFP-CapZ in AiCE-Tg mice

**Figure 1:**
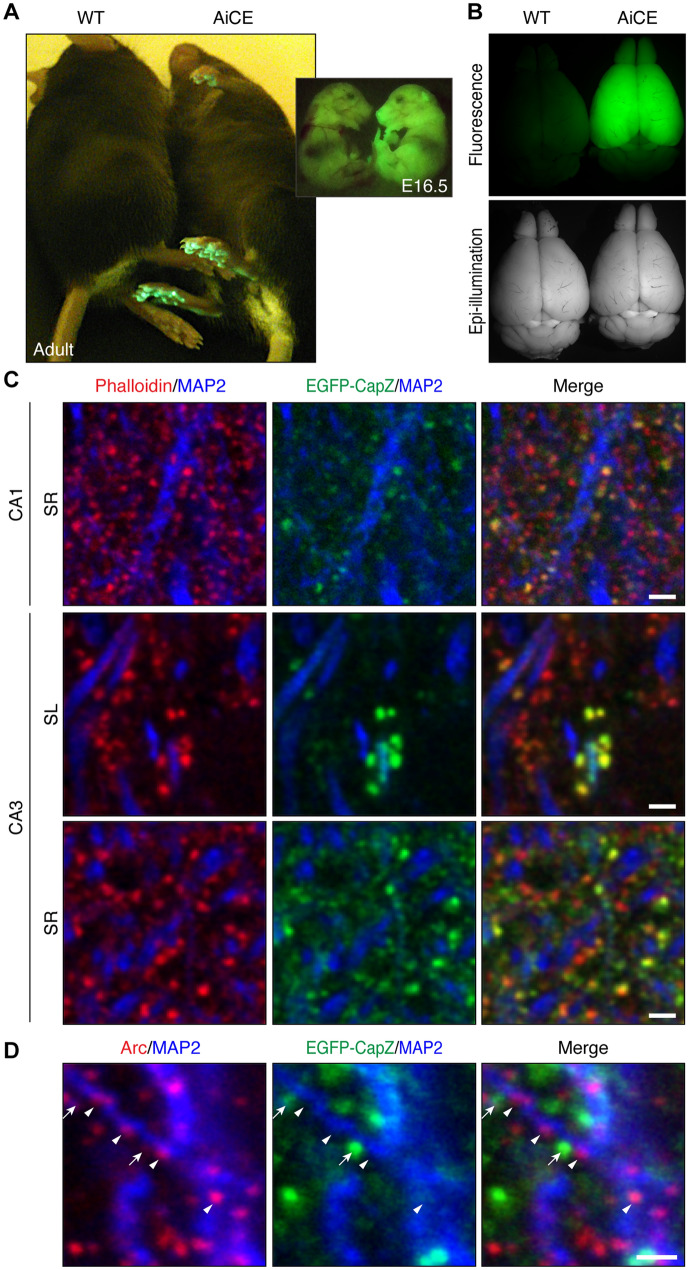
Properties of the AiCE-Tg mouse and transgene. (**A**, **B**) Visualization of EGFP. Whole bodies (A) and dissected brains (**B**) of wild-type (WT, left) and AiCE-Tg (right) adult or E16.5 mice under blue-LED light through yellow glasses as a long-pass filter (A and upper panel of B). (**C**) Non-homogenous localization of EGFP-CapZ (green) in spines with MAP2 dendrite maker immunolabeling (blue) and phalloidin spine marker (red) in hippocampal CA1 stratum radiatum (SR, upper), CA3 stratum lucidum (SL, middle), and CA3 SR (bottom). Scale bars, 2 µm. (**D**) Segregation from spine-like immuno-signals of Arc, which promotes AMPA-receptor endocytosis. Arc (red) and MAP2 (blue) immunolabeling with EGFP-CapZ signals (arrows) in CA1 SR. Scale bar, 1 µm.

For all of the experiments in this study, we used hemizygous AiCE-Tg mice carrying ~ 100 genomic copies of the transgene, a line that exhibits intense fluorescence (Fig. 1A and Supplementary Fig. S1A, B). Stable transmission was confirmed over tens of AiCE-Tg generations, with fluorescence in the footpad (Fig. 1A) and brain (Fig. 1B) bright enough to be detected with the naked eye through yellow filters under blue light from embryonic day 16.5 to adulthood. Additionally, corpuscle-like shaped EGFP-expression under the microscope, consistent with peripheral nerve expression, in the footpads of AiCE-Tg was useful for routine hemizygote selection within mouse cages (Fig. 1A). The exogenous fusion protein level in adult AiCE-Tg mouse brains was < 10% of endogenous CapZ protein levels (Supplementary Fig. S1C), and AiCE-Tg mice exhibited normal contextual fear conditioning (Supplementary Fig. S1D).

Punctate EGFP-CapZ signals were distributed across all cerebral cortex layers, overlapping with punctate phalloidin-labelled F-actin and rarely matched to dendritic shafts. The green/red (CapZ/phalloidin) intensity ratio varied widely, with bright signals in a minority of spines (Fig. 1C). Lucifer yellow injection into single neurons revealed CapZ content heterogeneity among individual spines within the same dendrite (Supplementary Fig. S1E). Prior findings have suggested a balancing role of Arc with respect to the promotion of AMPA-receptor endocytosis^3,25^. In this study, we observed Arc-immunopositivity in spine-like small puncta on dendrites, but those Arc puncta did not exhibit a pattern of preferential colocalization with EGFP-CapZ signals (Fig. 1D). These observations confirmed heterogenous EGFP-CapZ expression in the dendritic spines of AiCE-Tg mice.

### Relative increase in LTP-related EGFP-CapZ intensity mainly in spines

**Figure 2:**
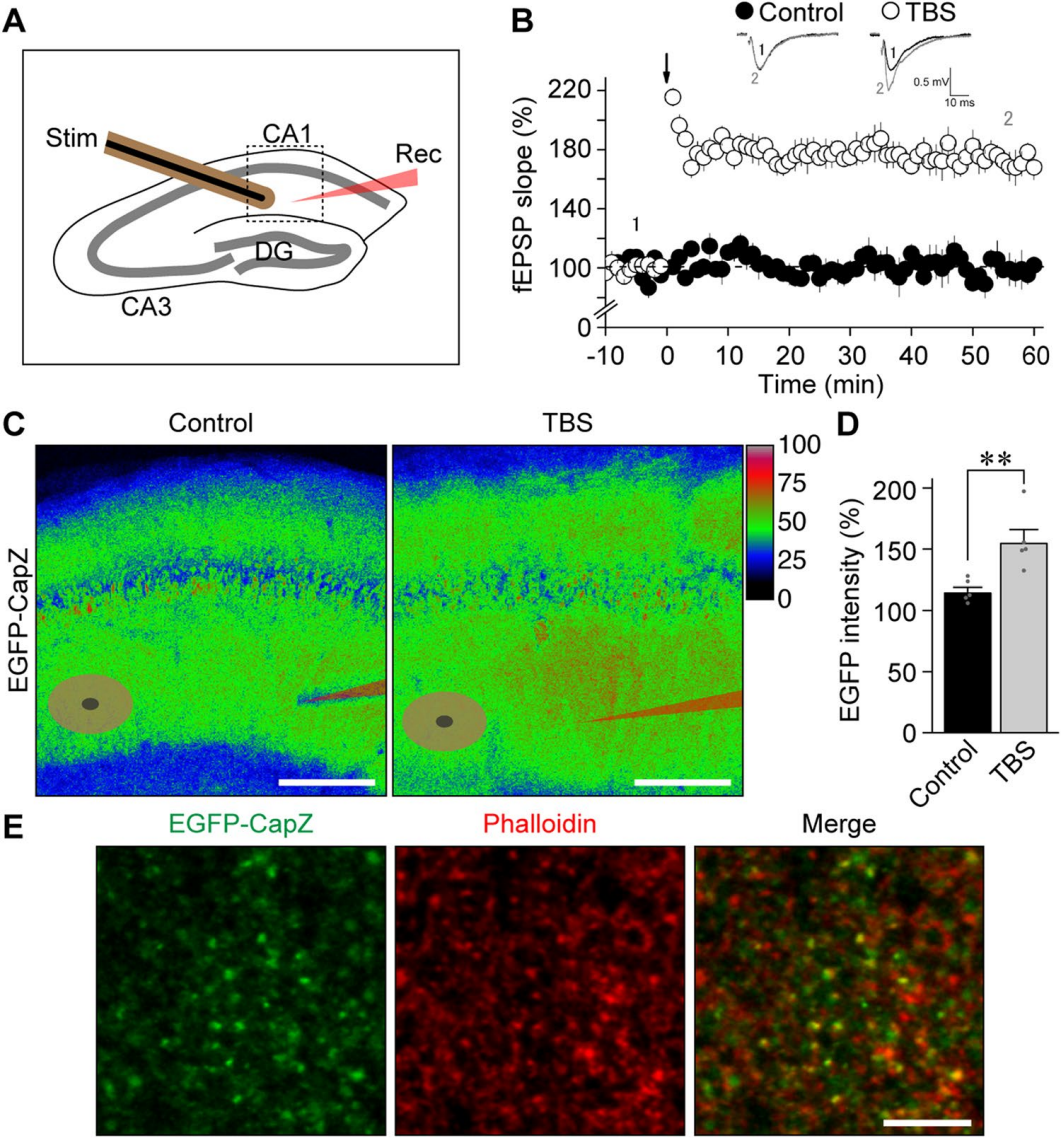
LTP-related increase in EGFP-CapZ fluorescence intensity mainly in spines. (**A**) Schematic drawing of AiCE-Tg acute hippocampal slice experiments. Theta-burst stimulation (TBS)-induced LTP was recorded in CA1. Concentric electrodes were used for stimulation (Stim) and field excitatory postsynaptic potential (EPSP) slope was recorded by glass electrodes (Rec). (**B**) LTP was induced at time 0 with TBS (open circles); there was no LTP in no-TBS controls (filled circles). (**C**) Representative images of EGFP-CapZ fluorescence in slices fixed immediately after the experiments (60 min after TBS). EGFP intensity (normalized to the highest intensity) is shown in pseudo-color scaling, with Stim (dual eclipse) and Rec (sharp edge) electrode sites. Scale bars, 200 µm. (**D**) Comparison of green fluorescence intensities of highest-intensity regions divided by that of neighboring regions within CA1. Bars are means with SEMs. n = 5 slices/group. **p = 0.0080 (Student’s *t-*test). (E) EGFP-CapZ in highest-intensity region. Large green puncta overlapped with phalloidin spine marker signals (red). Scale bar, 5 µm.

When LTP was induced in acute AiCE-Tg hippocampal slices (Fig. 2B) with theta-burst stimulation (TBS) via a fine concentric electrode that confines input current to the space between the black center and brown outer poles (brown mark in Fig. 2A,C), elevated EGFP signal areas were observed only in LTP-induced slices. EGFP-CapZ signal intensities of high-fluorescence regions were measured and normalized to neighboring low-intensity regions. Fluorescence was significantly more pronounced in high-fluorescence regions subjected to TBS stimulation than in control slices not subjected to TBS (Fig. 2D). Under high magnification (Fig. 2E), punctate EGFP signals were observed mostly within spine portions defined by phalloidin signals, consistent with an LTP-induction association.

### Procedures for quantitative image analyses of spines

**Figure 3:**
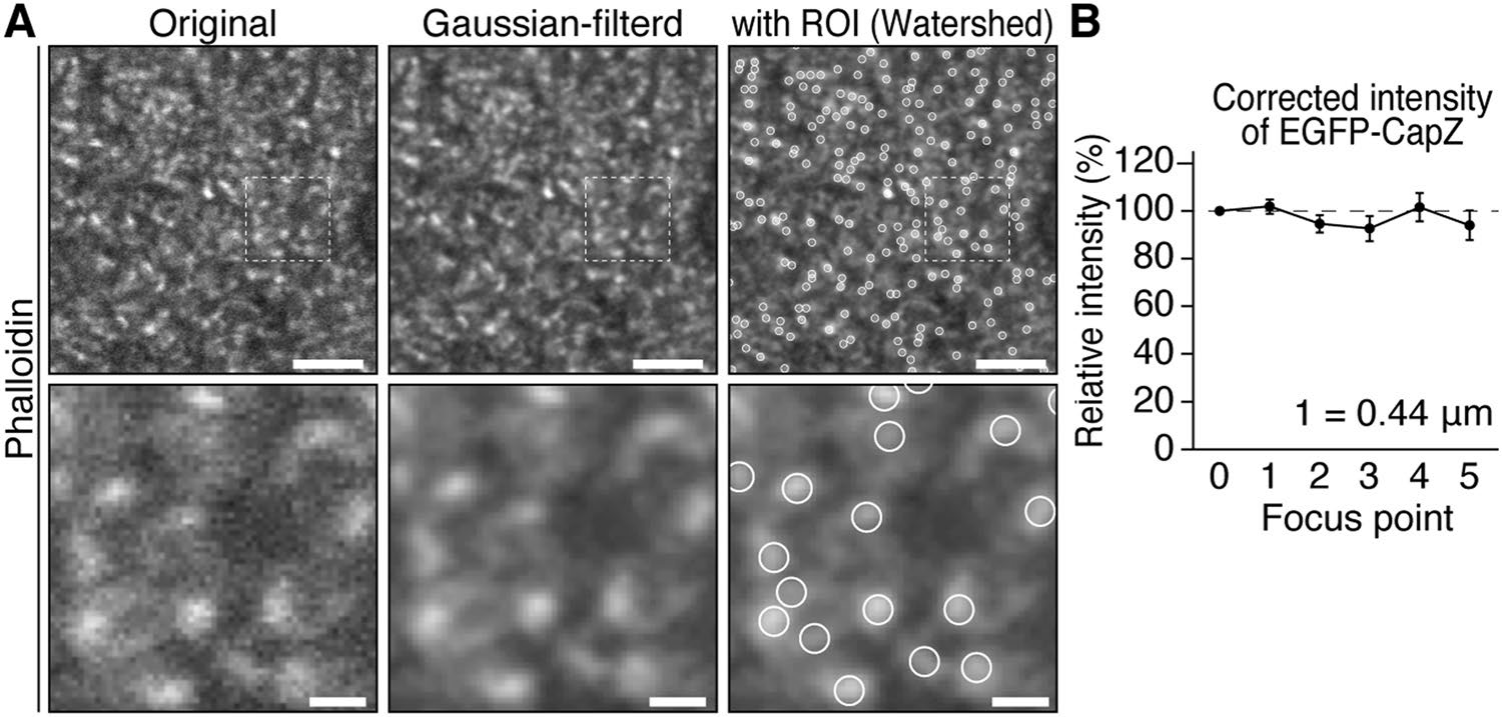
Procedures for quantitative image analysis of spines. (**A**) ROI setting procedures in TI workbench software^26^. Raw phalloidin signal data (left) were processed to get Gaussian filtered (3 × 3 pixel) images (middle) where ROIs correspond to individual spines (white circles, ~ 0.5 µm) were determined by a Watershed method (right panel). Intensity values for ROIs were taken from the raw data of each channel and analyzed. Bars, upper, 5 µm, lower, 1 µm. (**B**) Data fluctuation by focal plane. In each brain section from AiCE-Tg mice (N = 6), one part of cortex was divided into six viewing fields, and six images were taken of each field while the focal plane was moved from the surface to the interior (steps 0 to 5, 0.44 µm per step). EGFP-CapZ fluorescence intensities in spines were processed as described in the Methods. Corrected intensities (EGFP-CapZ intensity divided by phalloidin intensity) from these planes (values relative to that of the first plane) did not differ significantly across steps (*p* = 0.53, one-way repeated ANOVA).

**Figure 4:**
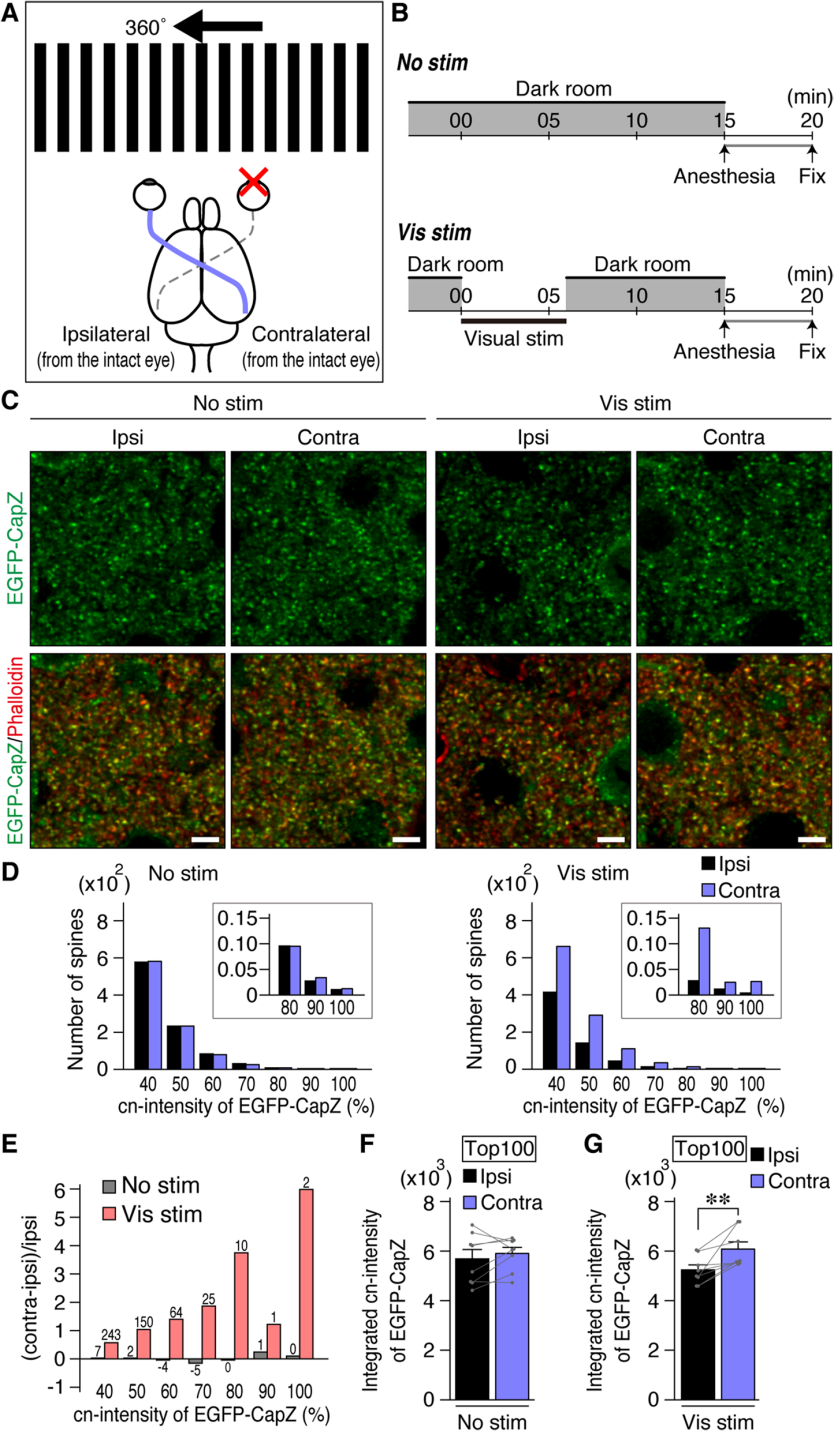
Brighter EGFP-CapZ signals in one side of the monocular region of primary visual cortex (V1M) after unilateral visual stimulus to AiCE-Tg mice. Schematic drawing of Vis stim (purple) to the contralateral V1M from a single intact (open) eye (**A**) and time course (**B**). AiCE-Tg mice had their right eyes covered with firmly glued patches and stayed in a dark homecage for 12 h, before being exposed to the visual stimulus (moving gratings in A, Vis stim) or kept in the dark (No stim). The mice were then anesthetized rapidly in the dark and their brains were fixed. (**C**) Two images were taken of layer IV of V1M from one coronal section from each animal on each side; Red, phalloidin spine marker; green, EGFP-CapZ. Scale bars, 5 µm. (**D**) Distribution histograms of cn(corrected and normalized)-intensities of EGFP-CapZ in spines from No-stim (left) and Vis-stim (right) animals, displayed from top to mid-range cn-intensity. Spine ROIs were defined automatically (Fig. 3A), then EGFP intensity was divided by that of phalloidin in the same ROI to obtain a corrected value for spine size and focal plane depth, c-intensity. For normalization of values, cn-intensities, from individual animals, c-intensities from spines were divided by the top-c-intensity (100%) in the included brain section (two images from both hemispheres). The average numbers of spine ROIs defined per image were (× 10^2^): No-stim contra/ipsi, 79/80; and Vis-stim contra/ipsi, 82/82. Histogram y-axis shows the average value per animal. (**E**) Contra-ipsi difference in spine EGFP-CapZ cn-intensity distribution. The ipsilateral side not given direct sensory stimulus was used as a basal reference state. Mean difference (increase) in the number of spines in the contralateral side was divided by that in the ipsilateral side to get ratio of difference (possible increment), and shown as bars in the same x-axis with the histograms in D. Inset number shows the average number of differences per image in a brain section. (F, G) EGFP-CapZ cn-intensities of largest 100 spines of each image were integrated, and the values were compared. N = 8/group; *p* = 0.25 Ipsi vs. Contra in No stim (**F**), **p = 0.0052 in Vis stim (**G**) (Paired *t-*test). Similar results were yielded, without phalloidin-correction (Supplementary Fig. S2B), by taking top 200 or 300 spines (Fig. S2A) and 6 h after Vis stim (Fig. S3).

For quantitative EGFP-CapZ intensity analysis, spines were labeled with fluorophore-conjugated phalloidin. Regions of interest (ROIs) roughly the size of a single spine (0.52 µm in diameter) were established at individual phalloidin signal clusters. The TI Workbench^26^ ROI selection algorithm (Watershed, see “Methods” section) enabled us to select most of the bright EGFP-CapZ puncta corresponding to probable spines, while excluding bright spots in somata and main dendrite branches (Figs. 3A, 4C, 7A, Supplementary Fig. S3A, S4D, S5B, and S6A). Because spine cross-sectional size depends on optical section depth relative to the imaged spine’s structure, EGFP fluorescence intensity (green) in each ROI was divided by phalloidin intensity (red) to correct for spine size and depth and then presented as corrected (c)-intensity.

**Figure 5:**
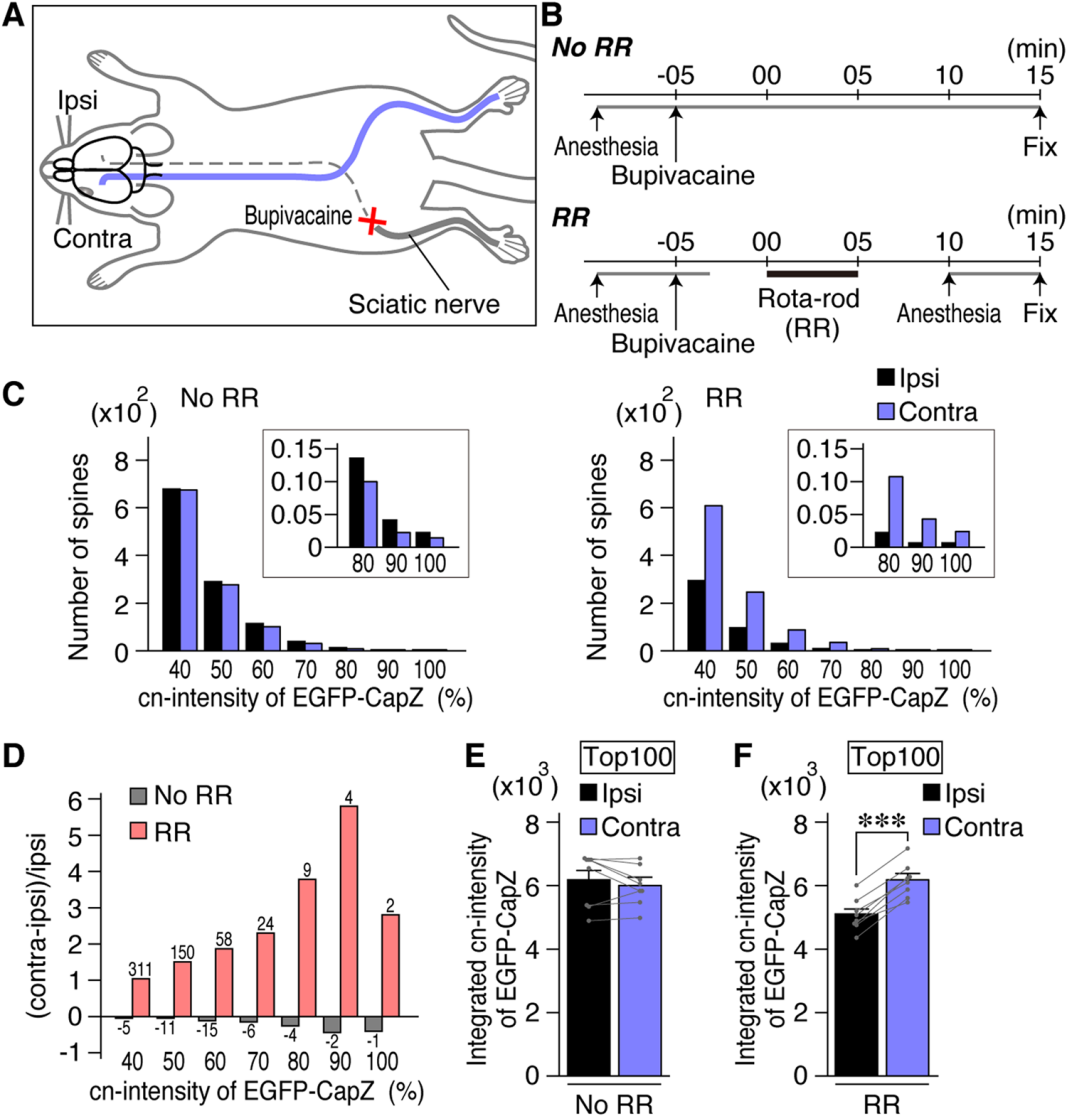
Brighter EGFP-CapZ signals in one hemisphere after unilateral sensory deprivation of AiCE-Tg mice subjected to rota-rod exercise (RR). Data are presented as in Fig. 4. (**A**) Schematic drawing of sciatic nerve inactivation with bupivacaine, a sodium channel inhibitor (confirmed by sensory tests in Supplementary Fig. S4C). (**B**) Time course of the experiments. Left sciatic nerve was quickly encapsulated by bupivacaine-soaked sponge sheet under anesthesia with the isoflurane. Mice were forced to do RR for 5 min or kept anesthetized (No RR) until sampling 20 min after sensory deprivation. (**C**) Distribution histograms of EGFP-CapZ cn-intensities in spines/animal from No RR (left) and RR (right) groups. The total defined spine ROI numbers per animal were (× 10^2^): No RR contra/ipsi 78/80; and RR contra/ipsi, 81/79. (**D**) Ratio of distribution difference of EGFP-CapZ cn-intensities in spines. (**E**, **F**) EGFP-CapZ cn-intensities of largest 100 spines in each image were integrated and the values were compared. N = 8/group; *p* = 0.35 Ipsi vs. Contra in No RR (E), ***p = 0.000035 in RR (**F**) (Paired *t-*test). See Figure S4A–B for location findings within S1, Figure S4C for vonFrey sensory test results, Figure S4D for images, Figure S4E for preliminary comparisons of c-intensities among experimental groups and wild-type animals and Figure S5 for sham-operated (saline instead of bupivacaine) controls showing no significant differences.

**Figure 6:**
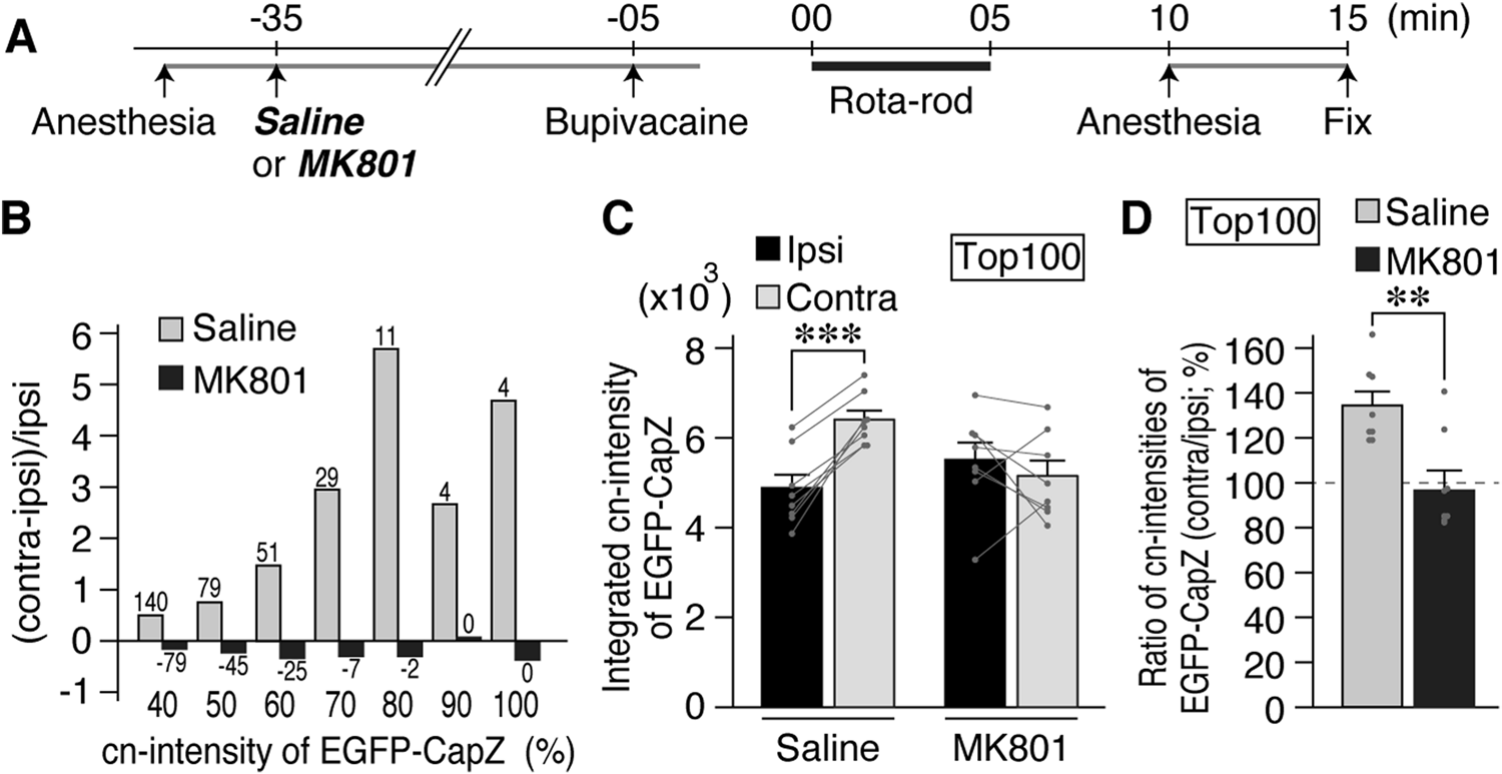
Left–right difference in EGFP-CapZ fluorescence was abolished by MK801. (**A**) Time course of experiments with MK801 (0.3 mg/kg, i.p., crosses blood–brain barrier) or saline injection prior to nerve inactivation and RR (behavioral task as in Fig. 5). (**B**, **C**) MK801 treatment abolished left(contra)- right(ipsi) differences in the RR group (compare to Fig. 5D–F, S5H) while the saline control group retained the RR- induced left–right difference. N = 8/group, ***p = 0.00024 Ipsi versus Contra in Saline treatment, *p* = 0.54 in MK801 (Paired *t-*test) in C. (**D**) Data in C were processed as contralateral/ipsilateral intensity ratios. These ratios from MK801-injected animals (right) were ~ 1.0 and differed significantly from those from saline-injected animals (left). N = 8. **p = 0.0046 (Student’s *t*-test) See Supplementary Fig. S6 for images and other data.

**Figure 7:**
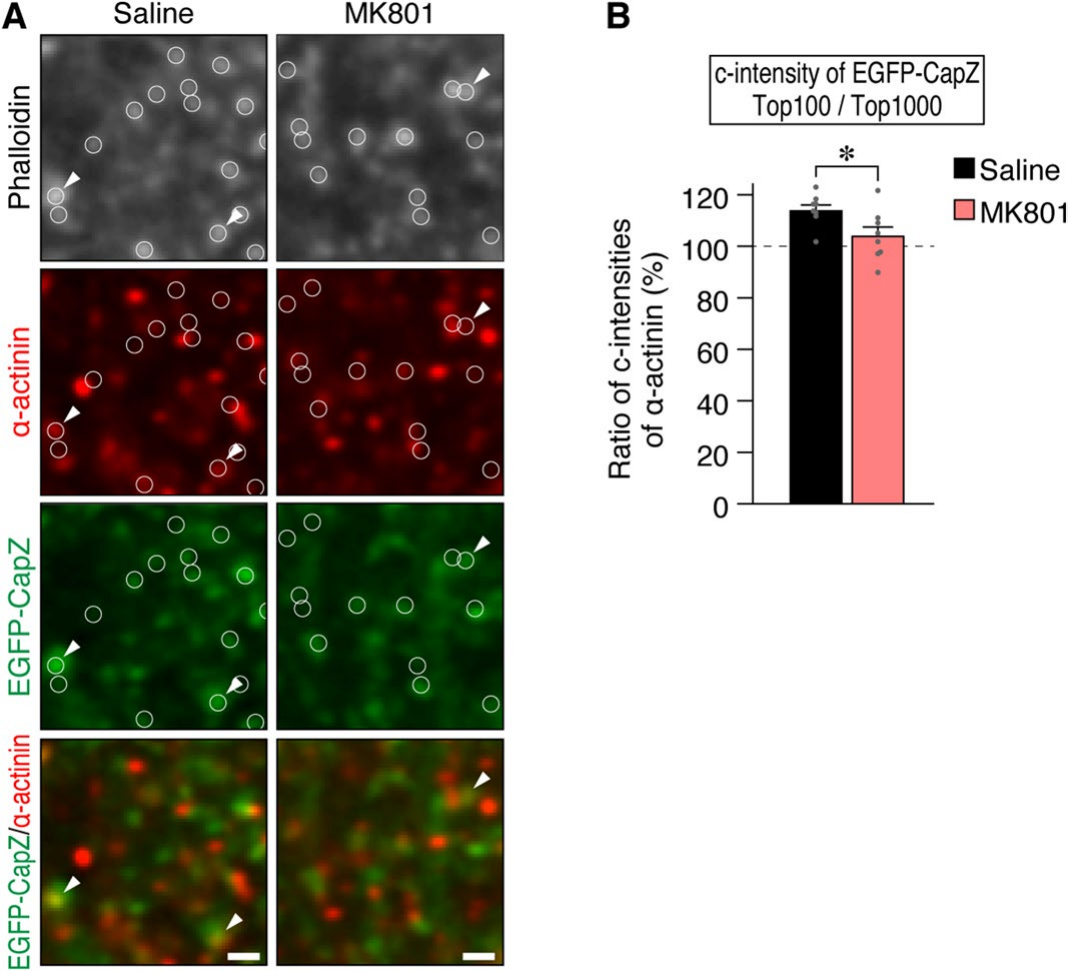
Colocalization of α-actinin with bright EGFP-CapZ fluorescence, abolished by MK801. (**A**) Pan-α-actinin antibody labeling and EGFP-CapZ in contralateral S1 of AiCE-Tg mouse with sciatic nerve inactivation after RR, with or without MK801 pretreatment. Representative images of α-actinin immunopositive puncta with some colocalization with EGFP-CapZ (small arrow heads). The spine-tops defined by phalloidin-staining are indicated with circles. (**B**) Some of larger α-actinin signals were found in spines with brighter EGFP-CapZ signals. The c-intensities of α-actinin were measured in phalloidin-defined spine ROIs (Fig. 3A). Values shown are ratios of α-actinin c-intensity in top 100/1,000 EGFP-CapZ c-intensities (see “Methods” section). In saline controls, ratios exceeded 1.0, suggesting preferential colocalization in spines with brighter EGFP-CapZ. MK801 pretreatment decreased ratio values significantly. N = 8/group; *p = 0.017 (Student’s t-test).

ROI c-intensity was stable across the range of focal depths used in this study owing to negligible variance of signal stability along possible slight changes in focal plane depth (Fig. 3B). For quantitative image analyses (Figs. 4-7, Supplementary Fig. S2-6), all procedures from brain dissection through data analysis were completed by researchers who were blinded to the groups (visually-stimulated vs. control, bupivacaine vs. saline, and saline vs. MK801). On average, phalloidin defined spines (8 × 10^3^ per image, 0.2 spines/µm^3^) accounted for about one-fifth of the number of spines (including thin filopodium) identified by electron microscopic analysis in the somatosensory cortex (1.07 synapses/µm^3^)^27^. Because spines with the largest physical dimensions and highest cytoskeletal F-actin levels should tend to have the most dense phalloidin signals, our selection of ROIs based on phalloidin brightness favored the inclusion of the largest (e.g. top 20%) spines, which fits well with previously reported CapZ distributions (25% of all spines, and 53% of large mushroom-type spines in hippocampal area CA1)^8^.

### Visual stimulation (Vis stim) increases numbers of spines with intense EGFP-CapZ fluorescence

We compared left and right visual cortices after delivering Vis stim to one eye in AiCE-Tg mice (Fig. 4A). In rodents, the medial monocular region of primary visual cortex (V1M) receives input from a single eye. Thus, it was feasible to compare the effects of monocular Vis stim between stimulated and non-stimulated sides of V1M in coronal sections. The unilateral visual stimulus (a moving grating pattern) was presented after a 12-h dark period to minimize background activities and lessen *Arc*-promotor activity^23^. Shortly before the dark period, a patch was glued firmly over one eye under anesthesia in dim light to minimize visual input via that eye during the operation. Immediately after 6 min of stimulation and 10 min of rest, the mice were anesthetized in the dark under an infrared scope; their brains were fixed 20 min after the Vis-stim onset (Fig. 4B).

Even without Vis stim (No stim group) after a 12-h dark period, clear EGFP-CapZ expression was observed in spines (Fig. 4C), suggesting that there is relatively persistent EGFP-CapZ distribution in some spines. The spines in animals in the Vis-stim group looked generally similar to those in the No-stim group, though a few brighter (more yellowish) puncta were seen in the stimulated (contralateral) side (Fig. 4C). For quantitative ROI analysis of EGFP-CapZ (green)/phalloidin (red) fluorescence ratios (c-intensities), we examined the strongest c-intensity regions among all spines in both V1M sides in each brain section from one mouse to obtain corrected-then-normalized (cn)-intensity percentages (normalized among animals relative to the top value, set as 100%, see “Methods” section). An EGFP-CapZ cn-intensity histogram for No-stim control animals (Fig. 4D) demonstrated similar intensity distributions on both sides. In Vis-stim mouse brains only, the V1M contralateral to the stimulated eye (i.e. in the stimulated V1M) included more spines with high cn-intensity than were present in the V1M ipsilateral to the stimulated eye (i.e. in the non-stimulated side of V1M). If one would like to define a number of “EGFP-positive” spines as a function of a specific intensity, a percentile threshold (e.g. in a histogram of percentile bins) can be applied as appropriate for particular experimental conditions. However, one should exercise caution in the normalization of values applied within each animal to compensate for inter-individual differences. Broadly distributed differentiation was observed from high- to middle-intensity spines (Fig. 4D), with the difference ratio being shifted toward high cn-intensity (Fig. 4E). The spine number analyzed here (X-axis of Fig. 4D and numbers of differences on the bar in Fig. 4E) was much smaller than the expected total spine number, 4 × 10^4^/image, calculated from electron-microgram observations^27^. When the 100 greatest cn-intensity spines (top 100) were integrated in each hemisphere, the stimulated (contralateral) hemisphere had significantly larger summed values than the non-stimulated side (Fig. 4F,G). This difference was maintained when the top 200 or 300 cn-intensities were summed (Supplementary Fig. S2A), as well as when the top 100 normalized EGFP-intensities without phalloidin intensity correction were analyzed (Supplementary Fig. S2B). Phalloidin intensity, which may reflect an aspect of spine status, did not differ significantly within the same Vis-stim group (Ipsilateral 0.57 ± 0.02, Contralateral 0.52 ± 0.02, N = 8, *p* = 0.26 in paired *t*-test).

To assess whether EGFP-CapZ expression might be affected by activity of the *Arc* promotor as seen in Arc-dVenus mice^23^, somatic EGFP-CapZ expression was measured. Somatic levels were similar across both V1M sides (Supplementary Fig. S2C), suggesting that *Arc*-driven EGFP-CapZ increases cannot explain the contralateral-versus-ipsilateral intensity shift that we observe within 20 min of stimulation.

Observation of sections from brains collected after a 2-h dark period following Vis stim had been conducted to locate the position of stimulus-dependent changes in EGFP-CapZ signals and had revealed marked increases in the stimulated side of V1M (Supplementary Fig. S2D). Because the difference extended from the rostral to caudal V1M (Supplementary Fig. S2E), a portion midway between the rostral and caudal ends was selected for coronal brain section analysis (Fig. 4).

Observations made in brains subjected to a 6-h dark period following the same Vis stim showed similar right-left differences in spines (Supplementary Fig. S2A–D). The contralateral-ipsilateral differences in 2-h or 6-h dark period groups would be expected to include Arc-promotor induced increases in the stimulated hemisphere. However, the contralateral-ipsilateral difference in the number of bright EGFP-CapZ spines at 6-h were similar to or, at most, doubled (up to a few thousand per 4 × 10^4^ spines in one image) the quantity of bright spines observed immediately after 20 min of stimulation. Hence, Vis stim changed EGFP-CapZ fluorescence intensity in a subset of V1M spines in AiCE-Tg mice and the change was observed to be maintained for at least 6 h.

### EGFP-CapZ fluorescence in AiCE-Tg somatosensory cortex was changed after unilateral sensory deprivation

Next, we examined the effect of sensory deprivation produced by unilateral bupivacaine (sodium channel blocker) treatment of sciatic nerve bundles (Fig. 5A,B). Sensory inactivation was confirmed with the von Frey test (Supplementary Fig. S4C) and sensory stimulation of the foot was achieved with rota-rod exercise (RR). EGFP intensity analysis conducted in layer IV of the hindlimb region of primary somatosensory cortex (S1) after the operation in mice subjected to RR yielded similar and more robust changes than were seen in V1M. Similar to the V1M data, some brighter fluorescent puncta were seen in the stimulated (contralateral) side (Fig. 5C for the graph and Supplementary Fig. S4D for images). A stimulation-related distribution difference was observed (Fig. 5D), with significant right-left changes in the integrated top-100 EGFP-CapZ cn-intensities in spines (Fig. 5E,F). Contralateral/ipsilateral intensity ratios from the anesthetized No RR animals were ~ 1.0, and those from the RR trained animals differed significantly from the ratios obtained for the control animals. (Supplementary Fig. S5H, N = 8. *p* = 0.000018, Student’s *t*-test).

The location to be analyzed was determined experimentally from within the somatosensory region in brain sections 24 h after unilateral sciatic nerve cutting, which produced a very clear difference in the place that corresponded to the lower limb region in the atlas (Supplementary Figs. S4A and B). There were no significant right-left differences in sham control mice that were treated with saline in place of bupivacaine (Supplementary Fig. S5A–G). Thus, it was confirmed that RR stimulation altered EGFP-CapZ accumulation in S1 spines.

### NMDA receptor blocker abolishes right-left EGFP-CapZ fluorescence difference

NMDA receptors are important mediators of learning-related plasticity. We administered an NMDA receptor blocker, MK801, to AiCE-Tg mice systemically 30 min before a unilateral-nerve inactivation experiment (Fig. 6A). Then the right-left difference (reproduced in saline pre-treatment controls) was abolished in MK801-treated mice (Fig. 6B–D, corresponds to Fig. 5D–F, S5H, and Supplementary Fig. S6A–B to Figs. S4D and 5C, respectively).

### Partial co-localization of α-actinin to bright EGFP-CapZ spines depends on NMDA receptor function

CapZ binds directly to the Z-disc protein α-actinin^28^, whose protein family has been proposed to modulate synaptic protein levels^29–34^, including postsynaptic levels of AMPA-type glutamate receptors via PSD95-anchoring^35^. Immunohistochemistry with a pan-α-actinin antibody showed partial α-actinin co-localization with EGFP-CapZ puncta in the stimulated side of S1 from mice used in the experiments shown in Fig. 6. Some α-actinin immunopositive puncta were colocalized with EGFP-CapZ signals (Fig. 7A, circles with arrowheads). Because quantitative comparison of immunolabeling across brain sections or counting after thresholding can be somewhat unreliable, we devised a method to compare values within image planes. Accordingly, we report the pan-α-actinin signal in phalloidin-identified spines (Fig. 3A) as c-intensities. Spines with the top 100 or top 1,000 EGFP-CapZ c-intensities within the same image were selected and α-actinin c-intensities inside were averaged; and the resultant values were divided to obtain a top 100/top 1,000-ratio of α-actinin c-intensities for that image (Fig. 7B, left black bar). Ratios > 100% indicated preferential localization of α-actinin in spines within the top 100 most-bright EGFP-CapZ signals. The ratio was decreased significantly by MK801 (Fig. 7B, right). Subsequently calculated 100/5,000 ratios (saline, 127.9 ± 3.94; MK801, 114.5 ± 4.06; *p* = 0.033) had greater values than the 100/1,000 ratios. Hence, α-actinin localization was more frequent and/or more accumulated in bright EGFP-CapZ spines, than in less bright spines, and this pattern was NMDA receptor current-dependent.

## Discussion

In the present study, we observed stimulus-dependent rapid changes in EGFP-CapZ fluorescence in dendritic spines in both the visual and sensory primary cortices of our newly generated AiCE-Tg mouse line. The most important point to note here, in the first paper reporting data from AiCE-Tg mice, is that changes in EGFP-CapZ reflect brain responses to relatively natural sensory stimulation and last for at least 15 ~ 20 min in vivo. To the extent that this phenomenon is related to the established notion of “neuronal plasticity”, it should be noted that LTP is just a parallelism (as shown in Fig. 2). There is no evidence-supported empirical rationale to limit consideration of the processes underlying brain plasticity to phenomena that have been well characterized up to now. Notably, the EGFP-CapZ differences were not seen in the presence of an antagonist of the NMDA receptor (Fig. 6), which is indispensable for some types of memory formation^36^. Although the detailed mechanism of EGFP-CapZ alteration is yet to be determined, our results provide a convergence of evidence suggesting that this traceable EGFP-CapZ fluorescence in spines represents an immediate plastic change in spines induced in vivo by lateralized inputs to the brain.

Given the rapid time course of EGFP-CapZ changes, accumulation and degradation should be considered as potential levers of CapZ regulation. An upregulation of protein synthesis is an improbable mediator of the post-stimulation changes in EGFP-CapZ documented here because 15 min after RR stimulation is too soon for transcriptional regulation, and also because neither the mRNA of CapZ beta2 nor the short *Arc* non-coding 5′ region incorporated in the AiCE transgene has a known localization signal for rapid local translation. In the context of constant CapZ generation and delivery that would be in active equilibrium with local degradation^37^, halting degradation would yield a rapid increase in the net level of the CapZ protein. Interestingly, CapZ contains a proteasome degradation signal that is thought to be hidden by specific binding proteins in heart muscle tissues^17,38^; hence, local neuronal CapZ levels could be upregulated by way of reduced degradation via unbinding of proteins that prevent degradation. Alternatively, CapZ accumulation within a specific spine might be consequent to CapZ relocation from surrounding loci. In addition to the known CapZ-binding proteins, newly generated barbed-end F-actin made by severing and branching should be considered as a candidate binding partner that enables EGFP-CapZ accumulation within a subset of spines^22^.

In our data processing for EGFP-CapZ intensities as relative values between contralateral and ipsilateral cortices, the nature of these potential changes is not limited to increases. That is, there remains a possibility that synapses in layer IV of the ipsilateral cortex receiving reduced input *lost* previously present EGFP-CapZ in that spine upon stimulation of the contralateral cortex. It would be prudent to consider both net increases and depotentiation-like decreases in EGFP-CapZ expression when interpreting observed changes in spines. When values not normalized for individual animals were compared, we did observe significant decreases in intensity between ipsilateral cortices of mice with versus without RR stimulation (Supplementary Fig. S4E; *p* = 0.0078 ipsilateral, *p* = 0.22 contralateral, Student’s *t*-test), even though no significant differences were found in multiple comparison testing (Bonferroni test). This observation may be in line with previous observations reporting small-spine reduction or shrinkage upon stimulation^3,7,39^, though the numbers of spine ROIs defined here by bright phalloidin were not altered substantially (see legends for Figs. 4D and 5C, possibly in the largest 20% of spines). While this issue is important to consider, we did not make comparisons across different animals in our analysis of the main results (e.g. between with versus without RR stimulation groups). We did not plan for such comparisons because once the brains become ex vivo, brain conditions can vary unnoticeably, such as during fixative perfusion. Experiments were performed in pairs (e.g. No stim and Vis stim) because the experimenter was blind to group designations, not because the groups were intended to be compared directly with each other. Further experiments using time lapse observation in vivo would be required to clarify the question of whether the experimental manipulations produce, in an absolute sense, increases and/or decreases in expression.

The aforementioned possibilities notwithstanding, there is substantial evidence favoring an LTP-like increase in EGFP-CapZ. Firstly, we showed previously that endogenous CapZ was increased selectively in LTP-induced layers^8^ and confirmed in the present study that EGFP-CapZ accumulates in some spines where LTP was induced in hippocampal slices from AiCE-Tg mice (Fig. 2). Although LTP in slices is not identical to synaptic enhancement in an intact brain, our data show that increases in EGFP-CapZ accompany burst stimulation sufficient to induce LTP and that weak inputs, such as those used for test stimuli in recording, have a milder effect on EGFP-CapZ. Insofar as this is true, EGFP-CapZ accumulation may be related to synaptic enhancement in vivo. The α-actinin protein, which colocalized with bright EGFP-CapZ signals in spines in our study, has long been related to synaptic plasticity^41,42^ and has been reported to bind and recruit PSD95^35^, a major AMPA-receptor anchoring protein. The presence of α-actinin supports the possibility of enhanced synaptic transmission in spines via PSD95 recruitment, wherein CapZ, a known binding partner in striated muscles, may guide α-actinin to specific spines. These results support the possibility that spines with both strong EGFP-CapZ fluorescence and α-actinin presence could represent spines with recently potentiated synapses.

Even if CapZ increases are due only to LTP-like changes, the number of changed spines in response to a given novel stimulus would be expected to be quite small. With approximately 4 × 10^4^ spines per image (1.1 synapses/µm^3^)^27^, ~ 1,000 biased EGFP-CapZ-positive spines (Figs. 4E, 5D) within an image represents involvement of, at most, a few percent of available spines. This portion is small enough to be consistent with a memory-related change based on theoretical models of stable memory circuit formation^43,44^. If EGFP-CapZ reduction contributes, even in part, to the right-left differences observed here, the net number of potentiated spines upon stimulation would be estimated to be much less in number.

Regardless of the reason for the changes, we observed many EGFP-CapZ signals even in mice without Vis stim for 12 h (Fig. 4C left No-stim images), with heterogeneity among spines. If these signals were induced in an input-dependent-manner before the 12-h non-stimulation period, binding proteins for CapZ (including barbed end F-actin) should be retained longer than 12 h to maintain the EGFP-CapZ signals. Otherwise, these signals may be induced in the primary visual cortex without external visual stimulus in response to something like spiking activities for memory replay during sleep^45^ or consequent to homeostatic plasticity. On the other hand, we observed a stimulus-dependent obvious EGFP-CapZ difference between the right and left cortices (Supplementary Fig. S3) with a longer time course after stimulation (6 h post-Vis stim). This observation supports the possibility that EGFP-CapZ changes seen 20 min after transient Vis stim persists for a further 6 h in the dark, at which time the distribution of EGFP-CapZ signals in spines remained non-homogenous with similar numbers of spines and right-left ratios (Supplementary Fig. S3). Surprisingly, considering known strong Arc-promotor activity, the magnitude of the increase in EGFP-CapZ intensity at 6 h was small, at only up to twice that seen after 20 min. These observations might be explained by CapZ’s degradation properties in the absence of binding proteins^17^, with binding protein levels within spines being a limiting factor for protection. Time lapse observation in our bright AiCE-Tg mouse line, which was successful in our preliminary experiments, should be used to characterize the dynamics of stimulation-induced EGFP-CapZ changes.

Indeed, there are many possible applications for EGFP-CapZ as a marker of CapZ-associated spine alterations. For example, the simple co-localization of EGFP-CapZ with other candidate molecules for synaptic plasticity in brain sections, as was done for α-actinin in Fig. 7, could be used to coordinate relationships among them. Because EGFP-CapZ seemed to remain for > 6 h after stimulation (Supplementary Fig. S3), such analyses could associate the distant time course of co-localization between CapZ and other key molecules, such as the fast comer, phosphorylated Ca^2+^/calmodulin-dependent protein kinase II^46^, and late comers, such as brain-derived neurotrophic factor^47,48^. In addition, bright EGFP-CapZ could be a useful marker in optophysiology, optogenetics, studies with fluorescent probes, and structural plasticity research.

In conclusion, because stimulus-dependent, synapse-specific changes are thought to underlie memory formation, molecular changes, such as changes in EGFP-CapZ accumulation with spine-specific traceability, are of great interest for plasticity research with respect to spine remodeling, regulation of the expression of this and other proteins^34^, including AMPA receptors, and providing clues regarding yet unresolved mechanisms. Because the EGFP-CapZ signal itself represents a plasticity-like feature of molecular event in vivo, clarifying relationships among molecular actors with live-imaging in AiCE-Tg mice could provide information about classification and time courses of traditionally recognized forms of neuronal plasticity.

## Methods

Detailed methods and materials are described in the Supplementary material.

### Generation of transgenic mice

Our established AiCE-Tg mouse line has been made available at RIKEN BRC [No. 09711; C57BL/6N-Tg(Arc-tTA/egfp/capzb*2) 100 Mkyph] and at the Jackson Laboratory (JAX#035474) for academic and non-profit researchers. First, the transgene (Supplementary material; Sequence of AiCE-Transgene.docx, Supplementary Fig. S1A) was constructed with cDNA of CapZ beta2 from mouse brain obtained by RT-PCR, Arc-7k promotor (a kind gift from Prof. Yamaguchi^23^), pTet-off system for tTA (GenBank: KY053478.1), synthetic linker sequences for P2A-self cleaving peptide, and pEGFP-C2 vector for EGFP (Clontech). Then six transgene-positive lines in 42 pups from 225 eggs were obtained. After transgene genomic content was analyzed (approximately 1, 3, 10, 10, 10, and 100 copies) by Southern blotting (Supplementary Fig. S1B), ~ 100-copy line hemizygotes (former #53; eggs preserved in RIKEN CDB L711~L715) were used in all experiments. Genotype was confirmed by PCRs of ear DNA with a three-primer mix, including primers for C-ter2 (CGC TTA AGA ACG ACC TGG TG) and pArev2 (CAA ACC ACA ACT AGA ATG CAG TGA) yielding a 282-bp transgene product, and the C-ter2 with CapZ3′non-code primer (CCA GAG GAG GGT GGT TAT CGG ACT TTA T) yielding a 232-bp wild allele product, separated in a 2.5% fine agarose gel, or by footpad-observation with 465-nm LED light and 500-nm-long pass glasses (Handy blue pro plus, RelyOn Ltd.) with no genotype segregation. Female 8–12-wk-old mice were used except for Fig. 2, LTP experiments.

### LTP induction and image analysis

Acute transverse hippocampal slices (300 µm thick) were prepared^49^ from 22 to 30-d-old mice. Field potential recordings were made with a patch pipette filled with 1 M NaCl. Schaffer collaterals were stimulated with a concentric electrode (FHC CBABD100, 100-µm diameter with 20-µm electric inner pole diameter). After 10 min of stable baseline recordings, LTP was induced by a TBS protocol^50^, comprised of a series of ten 5-pulse bursts (100 Hz pulse, 200-ms interburst interval) delivered five times every 10 s. After recording for 70 min, slices were soaked in 4% paraformaldehyde for 15 min, washed and then soaked in CF594-labeled phalloidin (1:1,000, Biotium) overnight on a shaker in a cold chamber. The phalloidin staining was used for framing and focusing in confocal microscopy, and Z-stack images of EGFP signals were collected over the slice thickness from the surface to obtain one maximum-projection image of each slice. Fluorescence intensities of possibly stimulated regions in the CA1 stratum radiatum were measured as mean intensities of four ROIs (40 × 40 µm^2^, set aside from visible vessels) in the highest region divided by the intensity in a neighboring CA1 region after background subtraction (mean intensity in CA1 stratum lacunosum-moleculare).

### EGFP observation in brain sections

Importantly, we kept the delay from diaphragm cutting to ventricle perfusion of paraformaldehyde under 30 s. For that purpose, before administering the fixative solution, 1 ml of PBS in a jointed short tube with a T-shape stopcock was delivered to provide a quick (~ 0.1 min) rinse of the vessels and thus to reduce autofluorescence and background reactivity from blood components. Gravity-driven fixation (120 mmHg, ~ 8 ml/min) was performed to keep blood vessel width within a natural range. Unfrozen brains were sectioned (100 μm) with a microslicer (DTK-3000 W, Dosaka). Cortical layer IV was determined by VGluT2-immunostaining (a presynaptic marker of thalamo-cortical projection. V1M or S1HL was first found as the medial side of VGluT2 immunopositivity, guided by the brain atlas). To avoid photobleaching during confocal microscopy with an oil-immersion 60 × objective lens (Olympus, NA 1.42), we used Alexa647 (relatively photostable fluorophore)-phalloidin for focusing while blinded to the (target) EGFP channel. Data analysis was performed in TI workbench^26^. The center of round ROIs (5 pixels = 0.515-µm diameter) was determined by segmenting Gaussian-filtered (3 × 3) phalloidin images with a Watershed algorithm^51^. Round ROIs (5-pixel diameter) were assigned to each part centered on the pixel with maximum brightness. Fluorescence intensities were calculated by averaging pixel values within each ROI without any filter (such as smoothing). The background value was set as lesser one of two mode values of image (of right or left hemisphere). EGFP-CapZ fluorescence (green) intensity in each ROI was divided by phalloidin (red) intensity to obtain a c-intensity. For each animal, one maximum c-intensity from the right or left hemisphere was used for normalization (100%) of all other c-intensities in both hemispheres to get cn-intensity.

### Monocular Vis stim

Vis stim was given in a quiet white insulated box (80 lx) with a monitor camera to confirm head movements. The night before, the right-eye of pentobarbital-anesthetized mouse was covered with a large eyepatch (a light-shielding sheet; perfect shielding grade, Oriji-Co., Gifu, Japan) glued with strong adhesive to dermis exposed with depilatory cream. The mice were placed in a dark room neighboring the insulated box for 12 h and given Vis stim of six pairs of 30-s moving gratings, with 30-s gaps, for 6 min. An open-top transparent column (diameter, 30 cm) with printed black standing bars was placed over a transparent chamber and the column, guided by a round rail, was rotated to make the bars move at 0.5 Hz from right to left (0° angle, square-wave gratings, spatial frequency of 0.05 cycle per degree, 10°/s) by a thread reeled by a quiet motor situated far outside of the box. Frequencies and other parameters were set to maximize neuronal responses in V1 according to previously established visually evoked local field potentials^52^. The rotation timing was controlled automatically by FZ software via a control box (DC-SB, O'hara’s system).

### Sciatic nerve inactivation

We employed a novel sciatic nerve inactivation procedure derived from a chronic constriction injury model^53^. Mice were anesthetized with isofluran (1.5–5%, Pfizer) and the left sciatic nerve trunk was exposed at the mid-thigh with a < 10-mm incision of skin. The nerve was cut (for Supplementary Fig. S4) or treated by encapsulation with a gelatin sponge sheet (Spongel, Astellas Pharma, cut to 5 mm × 5 mm × 1 mm) soaked in bupivacaine (20 μL; #B8262, LKT Lab, 0.5% in saline) or saline. The scar was quickly closed with surgical adhesive. The total operation time was < 5 min (~ 3 min on average). Five minutes later, mice were forced to exercise on a Rota-rod machine (25 rpm/min, Ugo Basile) for 5 min with experimenter support. The experiments for RR and noRR were done separately in order to keep blindness of experimenters to the groups; bupivacaine (Fig. 5) versus saline (Supplementary Fig. S5). When experimentally indicated, MK801 (0.3 mg/kg, *i.p.*) or saline was injected 30 min prior to the operation^47,54^.

### Statistical analyses

Statistical analyses were performed in Microsoft Excel, or SPSS version 20. Significance was assessed by independent *t-*tests, paired *t-*test, Mann–Whitney U test or analysis of variance (ANOVA) and Bonferroni’s test. Means are reported with standard errors of the mean (SEMs).

### Raw data in open science framework

Raw data, including our preliminary in vivo and cultured neuron results, are available at https://osf.io/8we4s/?view_only=15d5108e2a83478d8c7a6cd8cae4903b.

### Ethics statement and experimental animals

All animal protocols were in accordance with relevant guidelines and regulations and approved by the Animal Care and Use Committee of the Kagawa School of Pharmaceutical Sciences, Tokushima Bunri University or of the University of Tokyo. Two to five sex-matched mice were housed in plastic cages ad libitum food and water. They were housed under specific pathogen-free conditions at 23 °C (50–55% humidity) with lights on from 8:00 a.m. to 8:00 p.m. and handled gently.

## Supplementary information

Supplementary Information 1.

Supplementary Information 2.
