# Supplementary material for: Traceable stimulus-dependent rapid molecular changes in dendritic spines in the brain
Source: Sci Rep. 2020 Sep 17;10:15266. doi: 10.1038/s41598-020-72248-4 (PMC7499203; doi:10.1038/s41598-020-72248-4)
Supplement: Supplementary file 1 — Supplementary Information 1. [file 41598_2020_72248_MOESM1_ESM.pdf]

Supplementary Information for Title:

Traceable stimulus-dependent rapid molecular changes in dendritic spines in the brain

Authors:

Kazuya Kuboyama, Takafumi Inoue, Yuki Hashimotodani, Takuya Itoh, Tohsuke Suzuki, Aya Tetsuzawa, Yosuke Ohtsuka, Ryo Kinoshita, Ren Takara, Tohru Miyazawa, Pooja Gusain, Masanobu Kano, Maki K. Yamada*

*Maki K. Yamada, PhD

Email: makiky-tky@umin.ac.jp

This file includes:

- Supplementary text for methods
- Figures S1 to S6 with legends

Another Supplementary material: Sequence of AiCE-Transgene

Methods in detail

Immunohistochemistry and EGFP observation in brain sections

For fixation, mice were anesthetized with pentobarbital (50 mg/kg, Kyoritsu-Pharm) and perfused transcardially (120 mmHg, ~8 ml/min) with ~30 ml of freshly made 4% paraformaldehyde in 0.1 M phosphate buffer (pH 7.4). Before administering the fixative solution, 1ml of phosphate buffered saline (PBS; 8.1 mM Na_2HPO_4, 1.4 mM KH_2PO_4, 137 mM NaCl, and 2.7 mM KCl at pH 7.4) in a jointed short tube with a T-shape stopcock was delivered to quickly (~0.1 min, to keep delay within 0.5 min) rinse vessels to reduce autofluorescence and background reactivity from blood components. Brains were removed and put in the same fixative for 15 min. Unfrozen brains were sectioned (100 μm) with a microslicer (DTK-3000W, Dosaka). For one experiment (Fig. 1C, D), brains were placed successively in 5%, 15%, and 30% sucrose in 0.1 M phosphate buffer until they sank (≥ 12 h), sectioned (50-μm) with a cryomicrotome (Leica), and placed in PBS containing 1% Triton X-100 for 10 min. After blocking with 5% normal goat serum for 1 h, sections were incubated overnight at 4 °C in immersion buffer with the following antibodies: anti-MAP2 (chicken, 1:3000, Phosphosolutions;1100-MAP2), anti-Arc (rabbit, 1:1000, Synaptic Systems; Cat#156003). After rinsing in PBS, the sections were then incubated with fluorophore-conjugated secondary antibodies (Alexa594- or Alexa647-, 1:1000; Thermo Fisher, mixed with labeled phalloidin (0.2 U/ml, CF594- Alexa594- or Alexa647-labeled, Biotium or Thermo Fisher) in the PBS for 2 h at room temperature. Images were captured with an FV1000 or FV1200 confocal microscope (Olympus). Displayed images were processed with a 1×1-Gaussian filter in imageJ/Fiji for figures (except for Fig. 3). EGFP images for Figures S2C and S4A were taken with an

Orca Flash 4.0 camera (Hamamatsu photonics) mounted on a stereoscopic microscope (SteREO Lumar V12, Zeiss) equipped with a blue-LED light (465 nm, RelyOn Ltd.) for an emission light. ImageJ/Fiji was used for quantification of images except for the spine analyses.

LTP induction[1]

Acute transverse hippocampal slices (300 µm thick) [2] were prepared from 22–30-d-old mice. Mice were decapitated under isoflurane anesthesia, their hippocampi were isolated, and slices were cut with a vibratome (VT1200S, Leica microsystems, Germany) in an ice-cold solution containing (in mM): 215 sucrose, 20 D-glucose, 2.5 KCl, 26 $NaHCO_3$, 1.6 NaH_2PO_4, 1 $CaCl_2$, 4 $MgCl_2$, and 4 $MgSO_4$. Slices were transferred to a chamber perfused with the cutting solution at 33.5 °C. After 30 min of incubation, the cutting solution was switched to extracellular artificial cerebrospinal fluid recording solution containing (in mM) at 33.5 °C for another 30 min: 124 NaCl, 2.5 KCl, 26 $NaHCO_3$, 1 NaH_2PO_4, 2.5 $CaCl_2$, 1.3 $MgSO_4$ and 10 D-glucose. Slices were subsequently kept at room temperature for at least 1 h before recording.

Recording and stimulation electrodes were placed in the stratum radiatum. Field excitatory postsynaptic potentials were recorded with stimulus intensities that produce 30–50% of the maximum response at 0.05 Hz. Recordings were conducted with an EPC10 amplifier (HEKA Electronik, Germany) and performed at 28 ± 1 °C in a submersion-type recording chamber perfused at 2 mL/min with extracellular artificial cerebrospinal fluid.

Data analysis

Data analysis for spines was performed in TI workbench, doi:10.1093/jmicro/dfy015 http://inouelab.biomed.sci.waseda.ac.jp/inouelab-web/tiwb/TI%20Workbench.app.zip . For ROI setting, the segmented parts were filtered by intensity (highest pixel intensity of a part exceeded a threshold) and minimum size. The signal intensity threshold was set to F_{mean} + 2 × standard deviation, where F_{mean} was the average all pixel intensities of the image (20-pixel size threshold). For the frame selection, cortical layer IV was detected by VGluT2-immunostaining (a presynaptic marker of thalamo-cortical projection. V1M or S1HL was first found as the medial side of VGluT2 immunopositivity, guided by the brain atlas). VGluT2 immunostaining signals were maximized at the lower magnification. The sections were treated in 1% Triton-X100 for 10 min, 5% goat serum for 1 h, anti-VGluT2 (rabbit, 1:10000, Synaptic systems #135402) in 1% goat serum for 6–7 h at room temperature, then Alexa594-anti-rabbit secondary antibody together with Alexa647-labeled phalloidin overnight at 4 °C in 1% goat serum. The sections were mounted and coverslipped with Permafluor. When the maximal-intensity focal plane had any scratches (e.g. from sectioning), the focal plane was shifted up to two steps (< 1 μm) inside to the maximum, which would not affect the intensity readout (Fig. 3B).

For α-actinin immunohistochemistry, we used B-12 monoclonal antibody for C-terminal 20 amino acids (certified for α-actinin 1, 2 and 4; Santa Cruz #sc166524) and Alexa594-anti-mouse secondary antibody. No EGFP-signal bleeding was confirmed with secondary antibody control experiments (1.0 ± 0.0023 in top 100/1000, N = 4). Layer IV was determined by VGluT2 staining in an adjacent section. Immunofluorescence was normalized to phalloidin as a c-intensity.

Methods for supplementary figures

Western blotting

Following decapitation, brain proteins were extracted with 1% Nonidet P-40 in Tris-buffered saline (10 mM Tris-HCl, pH 7.4, 150 mM NaCl) containing 1× protease inhibitor cocktail (Nacalai tesque). Protein samples after centrifugation at 15000×*g* for 15 min were subjected to sodium dodecyl sulfate–polyacrylamide gel electrophoresis on 7.5–15% gradient gels (Nacalai tesque), followed by semi-dry electroblotting onto polyvinylidene fluoride membranes (Immobilon-FV, Millipore). After blocking with 10% SEA BLOCK buffer (no. 37527, Thermo) and 0.1% Triton X-100 in Tris-buffered saline, membranes were incubated overnight with mouse anti-CapZ β2 antibody (clone 3F2.3-s; 1:1000; DSHB) in 10% SEA BLOCK buffer in Tris-buffered saline, followed by Alexa 690-conjugated anti-mouse IgG secondary antibody (1:10000, Thermo Fisher). Labeling was detected with an Odyssey CLx Infrared Imaging System (LI-COR) as relatively ideal signals for quantification.

Contextual fear conditioning

An O'hara's commercial contextual fear conditioning system [white-walled sound insulating box (60 cm deep × 50 cm wide × 53 cm high) equipped with an overhead digital CCD camera and an electric-shock chamber (15 cm deep × 17 cm wide × 13 cm high)] was set up in a dim, quiet room (80 lux, 50 dB white noise). Each mouse was allowed to explore for 5 min before receiving one electric shock (2 s, 0.6 mA). The mouse was left in the chamber for additional 1 min. Freezing was measured 24 h after

training. Freezing time was determined from digital recordings automatically by FZ software (default settings), wherein freezing was <20-pixel change of body image (~5000 pixels) over 2 s (4 serial frames). We at first tried a common 3-shock protocol with no significant difference in the results in which the AiCE mice tended to spend more time freezing, then reduced the stimulus to 1 shock to avoid possible saturation.

Lucifer yellow injection and staining

Brains from PFA-perfused mice were postfixed in 4% paraformaldehyde in phosphate buffer, pH 7.4 for 1 h. Coronal sections (200 μm) were cut with a microslicer. Cells in CA1 (anteroposterior -2.30 mm from bregma) were injected individually with 4% Lucifer yellow (LucY, Sigma L0259) in water by hyperpolarizing current pulses (0.1 nA, 2 Hz) through an electrode under a microscope. The sections were incubated with anti-LucY (rabbit, 1:2000, Thermo Fisher; Cat# A-5750) and anti-GFP (rat, 1:1000, nacalai tesque; Cat# 04404-84) primary antibodies and then Alexa 594-conjugated anti-rabbit IgG (1:1000) and Alexa 647-conjugated anti-rat IgG (1:1000) antibodies.

Von Frey test

Each mouse was placed in a gray plastic tube (72-mm diameter, 90-mm height) with a mesh floor (5 mm spacing of 1-mm wires). The von Frey filament (4 g; Touch Test Sensory Evaluator, North Coast Medical) was applied 10 times to the plantar surface of the hind paw (inter-stimulus interval, ~5 s). The frequency of foot withdrawal was expressed as a percentage [(number of foot withdrawals per number of trials) ×100].

References

1 Lin, B. *et al.* Theta stimulation polymerizes actin in dendritic spines of hippocampus. *J Neurosci* **25**, 2062-2069, doi:10.1523/JNEUROSCI.4283-04.2005 (2005).

2 Hashimotodani, Y., Karube, F., Yanagawa, Y., Fujiyama, F. & Kano, M. Supramammillary Nucleus Afferents to the Dentate Gyrus Co-release Glutamate and GABA and Potentiate Granule Cell Output. *Cell reports* **25**, 2704-2715 e2704, doi:10.1016/j.celrep.2018.11.016 (2018).

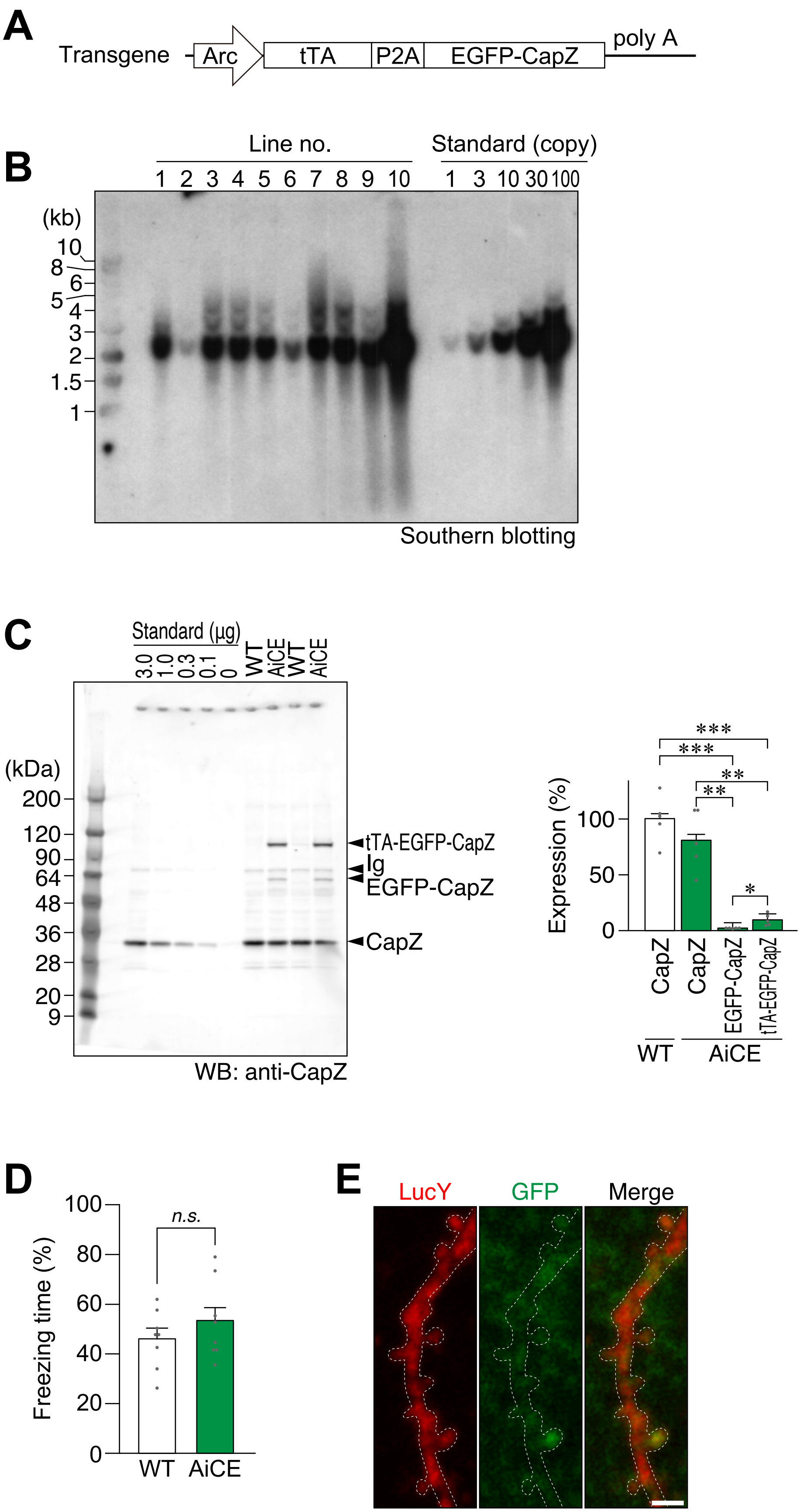
A
Transgene
Arc
tTA
P2A
EGFP-CapZ
poly A
B
Line no.
1 2 3 4 5 6 7 8 9 10
Standard (copy)
1 3 10 30 100
(kb)
10
8
6
5
4
3
2
1.5
1
Southern blotting
C
Standard (μg)
3.0 1.0 0.3 0.1 0
WT AiCE WT AiCE
(kDa)
200
120
90
64
48
36
28
20
9
tTA-EGFP-CapZ
Ig
EGFP-CapZ
CapZ
WB: anti-CapZ
Expression (%)
100
50
0
***
***
**
**
*
CapZ
CapZ
EGFP-CapZ
tTA-EGFP-CapZ
WT
AiCE
D
Freezing time (%)
100
80
60
40
20
0
n.s.
WT
AiCE
E
LucY
GFP
Merge

Figure S1. Supplemental data related to Fig. 1.

(A) Transgene construct used to create AiCE-Tg mice, including a 7-kb *Arc* promoter, cDNA of an EGFP-CapZ fusion protein with tTA, and a P2A self-cleaving peptide spacer. The sequence is available in another supplementary information. (B)Transgene Southern blot: lanes 1–10, mouse lines no. 1–10; lanes 11–15, XbaI-cut transgene DNA (2.4kb; 7179-9619 from 9875bp, coding tTA-EGFP-CapZ) used as standards for 1, 3, 10, 30, and 100 copies, respectively and for probe to XbaI-cut tail DNA. Tg-line no. 10 (firstly #53, ~100 copy) mice was used in this study. In these 10 lines, 6 lines (1, 3, 10, 10, 10, and 100 copies) were established and the eggs of 6 lines are kept frozen in RIKEN CDB (L695～L729). (C) Western blot with anti-CapZ β2 antibody (clone 3F2.3-s, DSHB) that binds endogenous CapZ and CapZ fusion protein(s) from the transgene in cortical lysate of AiCE-Tg(AiCE) and wild-type (WT) mice (left panel). The right panel shows the intensity of CapZ bands normalized to mean intensity of ~30 kDa signal (endogenous CapZ) from WT animals (means ± SEMs, N = 5/group, ~30 kDa signal of WT vs. AiCE-Tg, $p = 0.26$ with Student's *t*-test, $p = 1.00$ with Bonferroni's test). Long fusion protein tTA-P2A-EGFP-CapZ (uncleaved by P2A) detected in AiCE-Tg brains would similarly be included in the spine analysis, because tTA at the N terminus of EGFP would not differ from EGFP- CapZ in localization. *$p < 0.05$, **$p < 0.01$, and ***$p < 0.001$ (Bonferroni post hoc test following one-way ANOVA with $p < 0.05$). (D) Percent time spent freezing in 24 h after contextual fear conditioning (means ± SEMs, N = 7/group). n.s., not significant with Student's *t*-test. (E) Heterogeneously distributed EGFP-CapZ (green) in spines of CA1 dendrites (A neuron was filled with lucifer yellow, LucY, red).

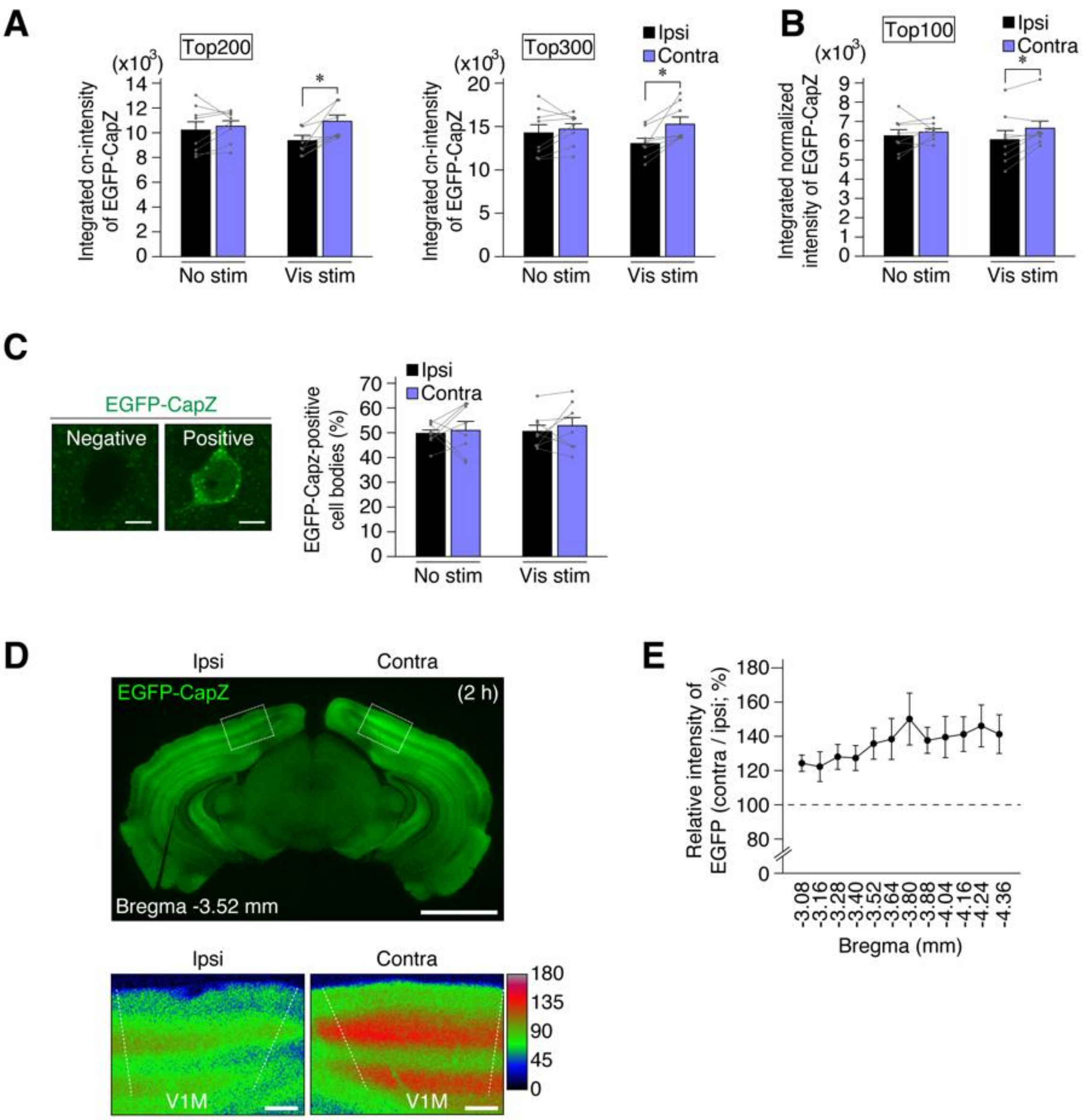

Figure S2. Supplemental data related to Fig. 4F.

(A) Integrated EGFP-CapZ cn-intensity from top 200 (left) or 300 (right) spines (means ± SEMs, N = 8/group). $*p < 0.05$ (paired *t*-test). (B) Similar integrated n-intensity of top 100 EGFP-CapZ signals without correction by phalloidin-intensity. N = 8/group; $*p < 0.05$ (paired *t*-test). (C) Ratio of EGFP-CapZ-positive cell bodies for estimation of expression level in right (Contra) or left (Ipsi) cortices 20 min after Vis stim or No stim. Mean percentages of EGFP-CapZ-positive neurons ± SEMs; N = 8/group, n.s., paired *t*-

test). Insets show representative images of neurons with (Positive) and without (Negative) detectable EGFP-CapZ. (D and E) Expression profiles of EGFP-CapZ in the brain 2 h after visual stimuli. AiCE-Tg mice with closed right eye, after 12 h in darkness, exposed to Vis-Stim for 6 min and then placed back in the dark for 2 h. (D) Representative images of EGFP-CapZ. Bottom images are boxed areas from the upper image in pseudo-color. Scale bars, 2 mm (top) and 200 µm (bottom). Ipsi, ipsilateral; Contra, contralateral to stimulated eye; V1M, monocular region of primary visual cortex, according to Paxino’s atlas. (E) Relative EGFP-CapZ fluorescence intensities in contralateral layer IV of V1M normalized to position-matched ipsilateral side (means ± SEMs, N = 7/group).

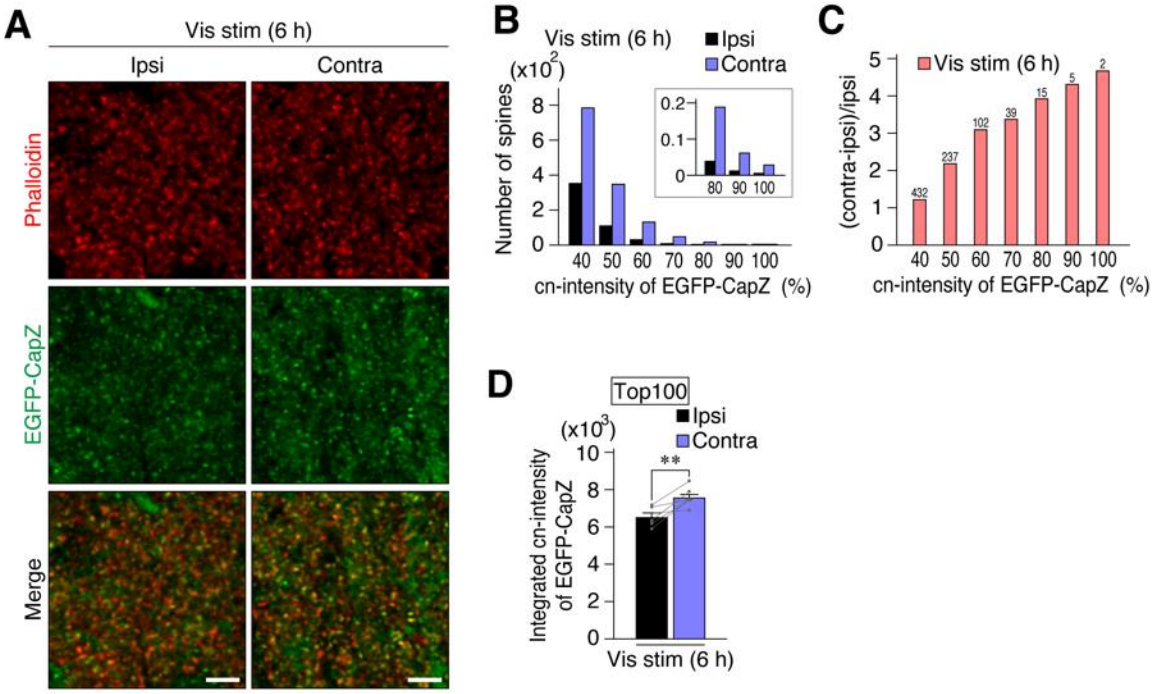

Figure S3. Supplemental data related to Fig. 4.

(A-E) AiCE-Tg mice with covered right eyes exposed to visual stimuli as described in Materials and Methods. After 6 h in a dark room, mice were sacrificed; brain sections were analyzed as in Fig. 4. (A) EGFP-CapZ (green) and phalloidin spine marker (red). Scale bar, 5 μm. (B) Distribution of EGFP-CapZ cn-intensity in top ranked spines. (C) Ratio of different distribution of cn-intensity of EGFP-CapZ in the contralateral to the intact-eye side normalized to ipsilateral (not stimulated) side (x-axis as in B). (D) Integrated EGFP-CapZ cn-intensity of top 100 spines (means ± SEMs, N = 6/group; $**p < 0.01$ in paired t-test).

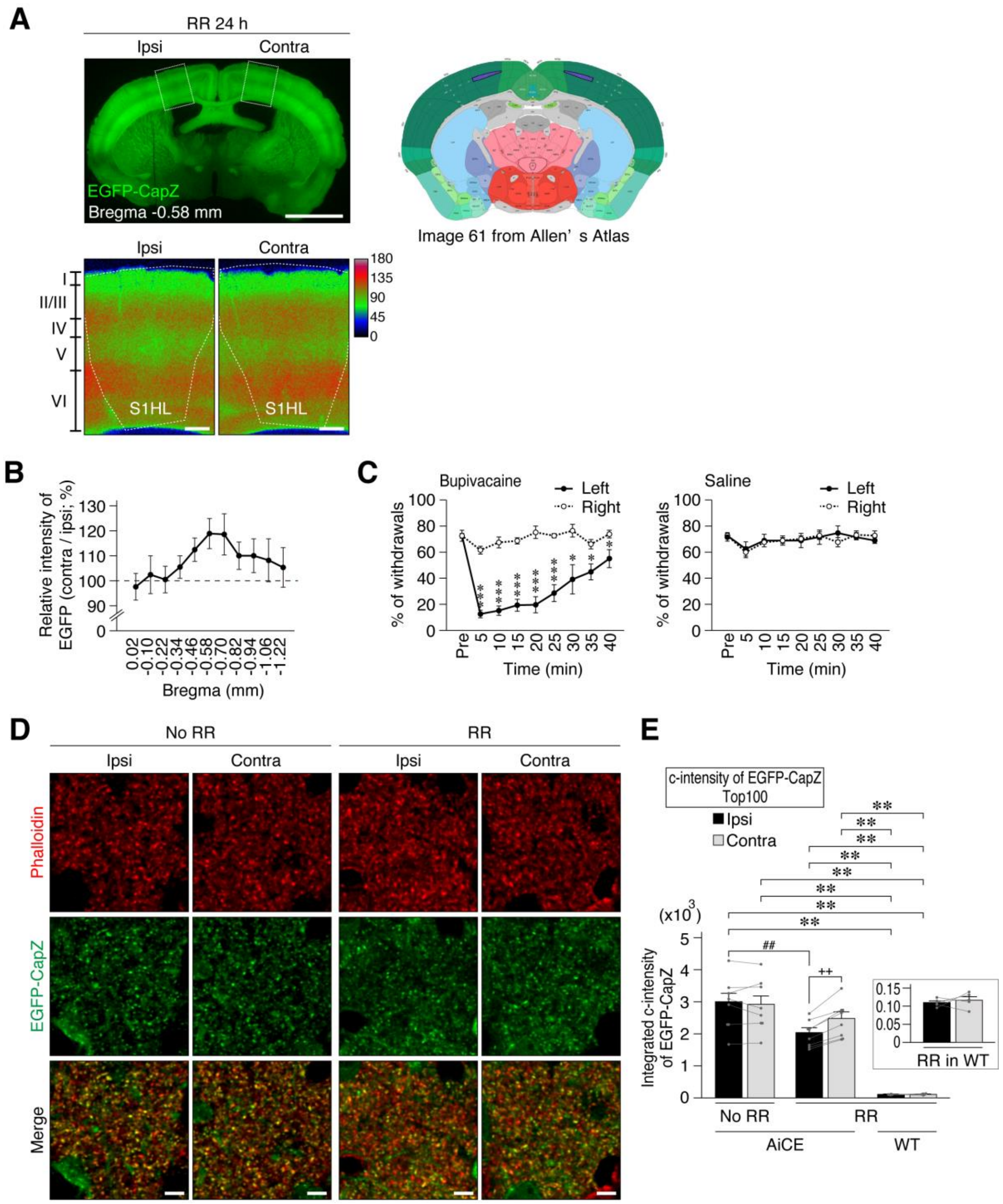

Figure S4. Supplemental data related to Fig. 5.

(A and B) EGFP-CapZ brain expression profiles in AiCE-Tg mice 24 h after left sciatic nerve cutting; unilateral sensory stimulation delivered subsequently by voluntary movement plus 5 min of RR 15 min prior to fixation. (A) Coronal section -0.58 mm from

bregma. Pseudo-color images (bottom) taken from boxed areas in the upper image. Scale bars, 2 mm (top) and 200 µm (bottom). Ipsi, ipsilateral and Contra, contralateral to nerve intact; S1HL, hind (lower) limb region of S1 found according to Paxino's (top) and Allen's (bottom, right) atlas. (B) Sequential coronal sections (100 µm thick) analyzed for relative EGFP-CapZ fluorescence intensities in contralateral layer IV of S1HL normalized to position-matched ipsilateral side (means ± SEMs, N = 6/group). (C) Sensory inactivation of left sciatic nerve in AiCE-Tg mice with saline or bupivacaine treatment; loss of sensation demonstrated by von Frey tests 5–40 min after the operation. N = 8/group. ***$p < 0.001$, *$p < 0.05$ in Mann-Whitney *U* test. (D) Representative images of somatosensory cortex layer IV used for Fig 5, at likely hindlimb region; position was experimentally determined in sections taken 24 h later (Fig. S4A, B) and VGluT2-immunostaining signals. Red, phalloidin-labeling of spines; green, EGFP-CapZ. Scale bars, 5 µm. (E) Integrated c-intensity without normalization from individual animals for the green channel signals (including flavoprotein or other autofluorescence also in the WT) from top 100 spines in AiCE-Tg used in Fig 5 or WT mice 15 min after 5 min of RR following left sciatic nerve inactivation (means ± SEMs, N = 8 in AiCE-Tg, N = 5 in WT). **$p < 0.01$(with Bonferroni correction), ##$p < 0.01$(t-test) and ++$p < 0.01$(Paired t-test for three pairs of ipsi and contra), $p > 0.05$ for other pairs for three types of statistics.

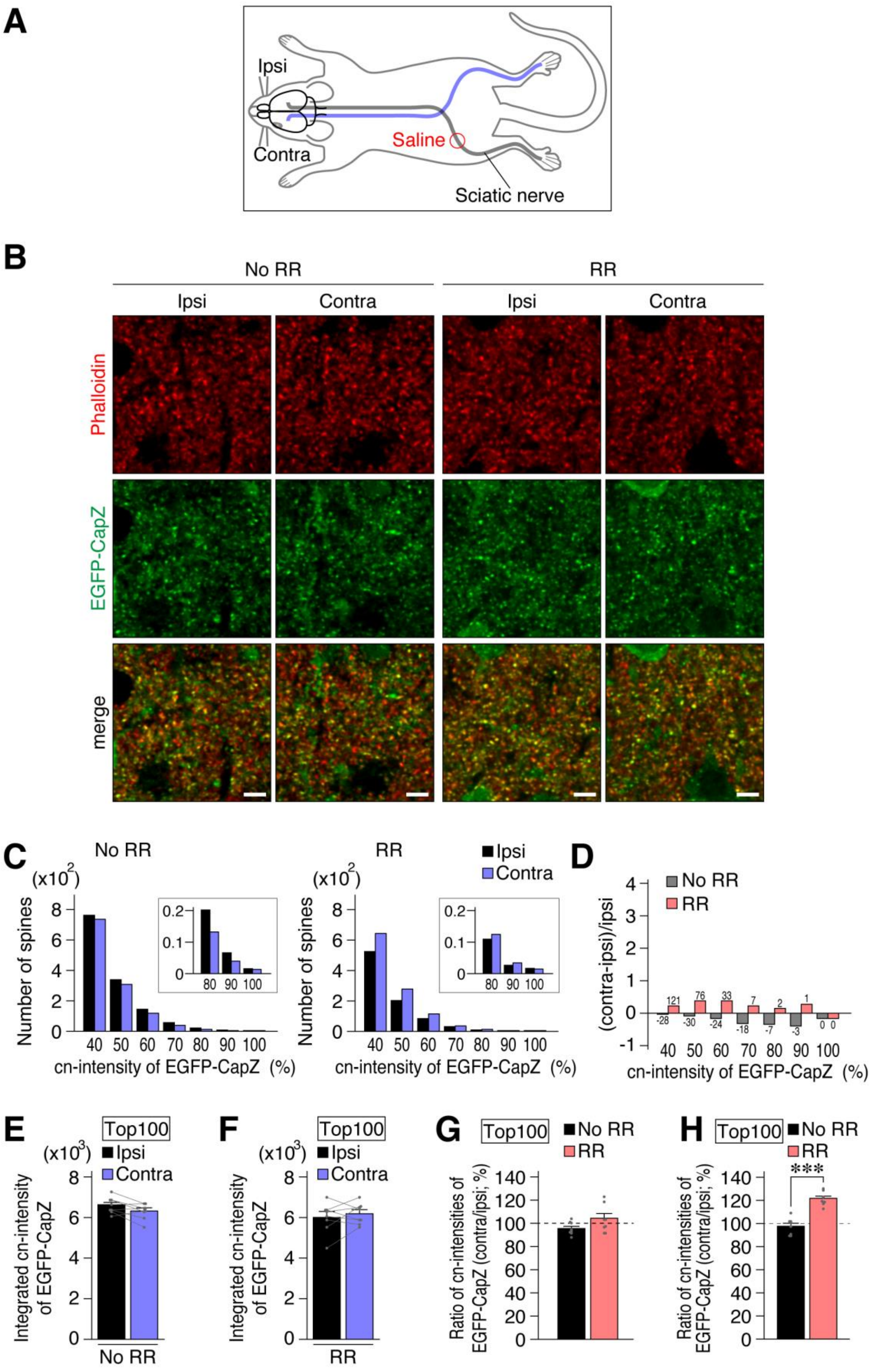
A
Ipsi
Contra
Saline
Sciatic nerve
B
No RR
RR
Ipsi
Contra
Ipsi
Contra
Phalloidin
EGFP-CapZ
merge
C
No RR
RR
Ipsi
Contra
(x10²)
Number of spines
cn-intensity of EGFP-CapZ (%)
D
No RR
RR
(contra-ipsi)/ipsi
cn-intensity of EGFP-CapZ (%)
E
Top100
(x10³)
Ipsi
Contra
Integrated cn-intensity of EGFP-CapZ
No RR
F
Top100
(x10³)
Ipsi
Contra
Integrated cn-intensity of EGFP-CapZ
RR
G
Top100
No RR
RR
Ratio of cn-intensities of EGFP-CapZ (contra/ipsi; %)
H
Top100
No RR
RR
***
Ratio of cn-intensities of EGFP-CapZ (contra/ipsi; %)

Figure S5. Additional supplemental data related to Fig. 5.

Sham-operated control data (saline instead of bupivacaine) presented as in Fig. 5 (A-F), and the ratio data of saline- (G) or bupivacaine- (H) treated AiCE-Tg.

(A) Schematic overview of the experiment. (B) image of EGFP-CapZ fluorescence (green) and phalloidin spine marker (red). Scale bar, 5 µm. (C) EGFP-CapZ cn-intensity distribution in top ranked spines. (D) Contralateral/ipsilateral spines ratios. (E, F) Integrated EGFP-CapZ cn-intensity of top 100 spines (means ± SEMs, N = 6/group; n.s. in paired *t*-test). (G, H) Relative EGFP-CapZ cn-intensity of top 100 in the contralateral side normalized to the ipsilateral side of saline-treated (G, N = 8/group. n.s.) or of bupivacaine-treated groups (H, N= 8/group. ***p = 0.000018, both in Student's *t*-test.)

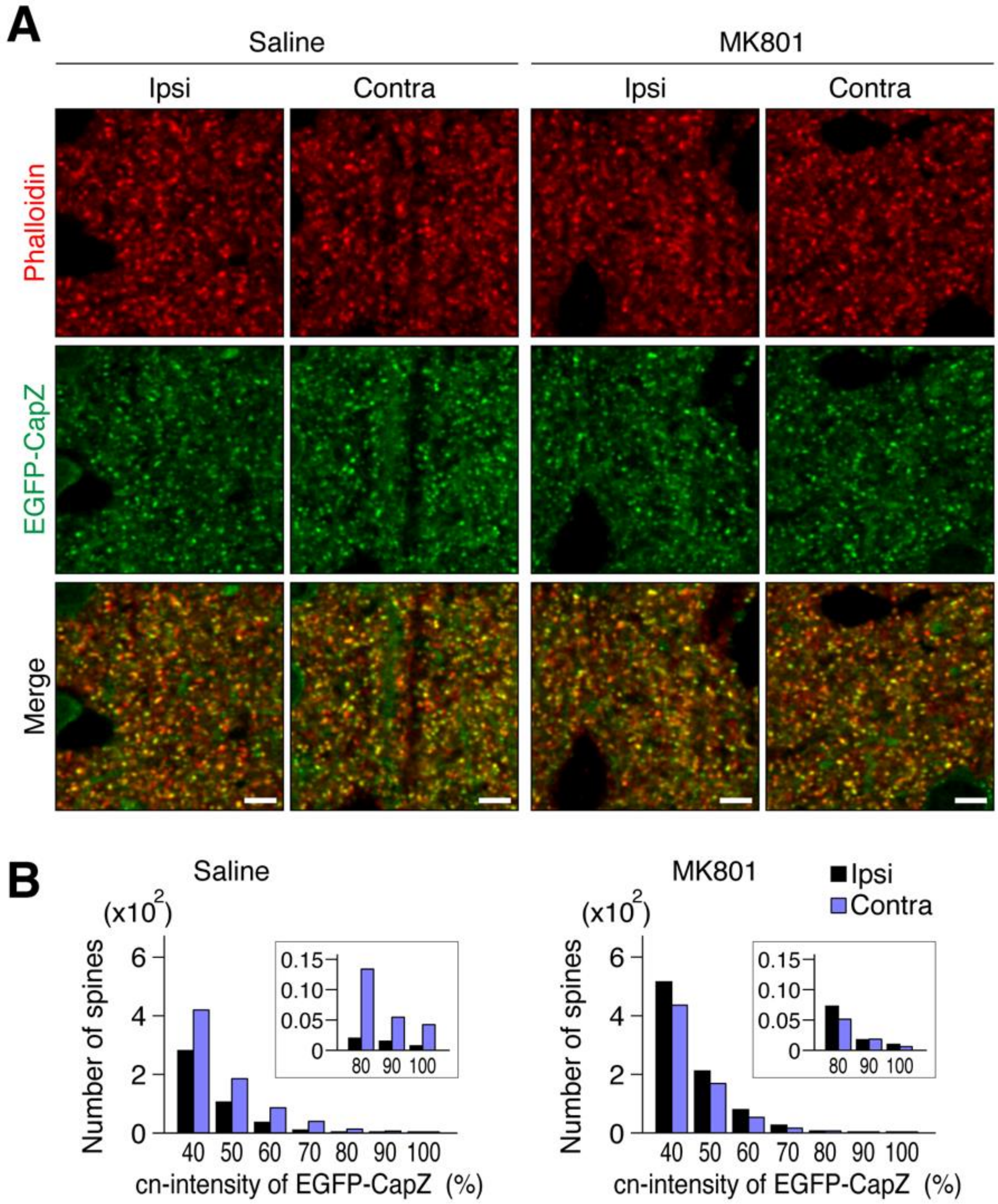

Figure S6. Supplemental data for Fig. 6.

Data are presented as in Figs. S4D and 5C. (A) Images of EGFP-CapZ fluorescence (green) and phalloidin (red) in mice after RR under sciatic nerve inactivation with saline or MK801 pretreatment shown in Fig6A. Scale bars, 5 µm. (B) Histograms of EGFP-CapZ cn-intensity distribution in spines.
