# Supplementary material for: Traceable stimulus-dependent rapid molecular changes in dendritic spines in the brain
Source: Sci Rep. 2020 Sep 17;10:15266. doi: 10.1038/s41598-020-72248-4 (PMC7499203; doi:10.1038/s41598-020-72248-4)
Supplement: Supplementary file 2 — Supplementary Information 2. [file 41598_2020_72248_MOESM2_ESM.pdf]

Supplementary material; Sequence of AiCE-Transgene for Title:

Traceable stimulus-dependent rapid molecular changes in dendritic spines in the brain

Authors:

Kazuya Kuboyama, Takafumi Inoue, Yuki Hashimotodani, Takuya Itoh, Tohsuke Suzuki, Aya Tetsuzawa, Yosuke Ohtsuka, Ryo Kinoshita, Ren Takara, Tohru Miyazawa, Pooja Gusain, Masanobu Kano and Maki K. Yamada*

*Maki K. Yamada, PhD

Email: makiky-tky@umin.ac.jp

This file includes: Sequence of AiCE-Transgene

Another Supplementary material: Supplementary text for methods and Figures S1 to S6 with legends

9869 bp Transgene SalI cut

TCGACTAGCGCTACCGG

GGGGGGCCATCTGCTTTCTCCTGGAACCAGAGCTCTAGGGTACAAGTAAACAAA
TACCTGATACAAGATCTTTGTGCAGGTCTGGTTTAAGAGTCACAAAGCCACCAAT
GGCAGGGACAATGTCACCAGTCTCCAGCCAGGGACTCTGGTCCCCACCTCTCCT
TCAGGGCAGGGGCTGAGCAGGTGTAGGGCAGGGTGGGCCCTGGGTGGTGGCAG
GCTCAGCGCACAGAGCCTTCCTGCGTGGGGAAGCTCCTTGCTGCGTCATGGCTC
AGCTATTCTCAGCCTCTCTCCTTTTATGGTGCCGGAAGCAGGCAGGCTGCTGCTG
GGCTGGCTCTGGGAGGTATTTAAATGGAATTGCAGAGAACAGCAGGAAAACAC
AGCCGCCAGGGTGGGGGTGGCAGCCTTGAACGCCACCCTCTAACTGGCATGGG
CATCTGAACTCTCAGCTCTGGGGGTGTCTCATCGGCCAGCTGGCAGTAGCCCCC
CCAACCCCACCCCTGATGGAGAACTGCTCCTGTATGATCCAGCCCAGTGGGCGC
ACAGGGAGTTGGTAGGTGGGCACTCAGACTGGCTAGGGCCCCAGTGGAGCAGA
GATGAGGGGTGGCTGCCAGCCTCCCACCTCAGCCCAGCCTCCCATGGCTCTTAC
TCATTCCTGGTTCGTAACTCTGCCCCTACCCCCAGCTCAGCACTGGCTACCTGGA
TATAGGGAACCCTAATAGTCTTCATTCTTGTCCCTTTGGTAGCCCAAATTCCTGGG
CCATCTCTCCAAAGCAGGGACAGCTCACACATCCAGAACACCAGCCACCAAATC
ATCTCCCCATATCCTGATTCTTTTCTACCCTCTCTACCCTCCATGTCCTCCCCAGGC
TGAGGAGGTCCTGGGTACAATGGCCCTGGCTCAAGGCTGGTGTCCTGGCTAGAA
TAAGGGAGACCTGACTCACCTTGATACCTGGTCTCTGCTACCATCCATGTTCTGC
CCCCACATGGACAAAACCCACATGGCCATGTCCTTGGCCTGGAGTAGCACCCTC
ACACATCTGGCAGTCCCTCTTTATGTCTGGCTGTTGGCTCCTGCATTCCAGTGAT
GGCTGGAAGCAGGTACACTAGGAAAAGCTAAAGGGGGCTACGACTGTGACATA
TATGGTTGCACACAAAACACATTTCCAGACCTGTGCAGATACCAAGATTGACCA
CTCATCTCAGAGACTCTGTGCAGATGCACACAAGATAGACCAAGGCACGCTGTG
CCTGGAGACCCTTCCTAGAGGCACAGAATATGGACAGCTATAGGGAGTCAAGGC
TTTCTCACTTTAACATAGAGTTGGCATTTGTCAACTGAACAGGAGGGTGGACAG
TTGGTCAGAAGGATCCTTGTAGCCACAGGTCCACCCAGTGCTTTCTCAGCCTTC
ATGAAGAAGAAAGAGGAGGAGGAAGAGGAGGAAGAGGAGGAGGAGGAGGAG
GAGGAAGAAGAAGAAGAAGAAGAAGAAGAAGAAGAAGAAGAAGAAGAAGA
AGAAGAAGAAGAAGAAGAAGAAGAAGAAGAAGAAGAAGAAGAAGAAGAAG
ACAAGGAAGAGGAAGAGGAGGAGGAAGGGGAGGAGGAGGAGGAAGAGGAGG
AGGTGCCCTAGTGGCCTTTTAGTACAGTCTGCCTGTTGTATAGTTGAGACCAAGG

TGCTGAATGAGTGCCATGAAGTCACACACAGTCTCAGGGAGATGCAAAAGTTCC
TGTCCACAGCGCTATGGAGGCTTAGGCTAGGCACAGACAGGCTATCTGACATGG
ACCCAAAAGGGGCTTCCTCAGGTCTAGCTCCTGTCTCTTGATTCTACACTTACGC
TGGCCCCCTAGTGTGTGAGTGCTGAGCTCTGTACCCACAATGCCTGCATGGCTTC
GCCTTCCTTGTTCTAGACTATTCTAGCTTGAGCAATGACAATTACATGGACTGCCT
GTTTTCTGCAAGAAGAATCCCCAGGAAGTGCTGGGTGCTCTTTGCCAAGTTCGG
AGGCTCTAAGCAAGGCCACCCACTGGCTGGCGCTGACAAGTTGCAGAAGTAAC
CAAGCTAGAAGCACAACAGAGGGCCATCAGGTGGGGAAAATGAGGCCAGGGA
AGGGGTGGGGACTTAGAGAGACTTACTCATGTTCCCATAATACAAGGGAGTGTC
TTACATAAGGACTCTAGGAGCCTGGAGGTCTCCGTCTGACCTCTAGCCCAATTCC
TAGCCTCTATCATTGTGTGACTTGGATAGGTTTCTGGCCCTCCGTCCCCCTTATGA
ATCATGGCTGGGACCAGGGAGGAACACAGGTCTCTTGAGGACAGACTCCCACA
AACCTGTGTCCTTGTCTACAGTCAGGTGGGCAGTGGGAAATGCCTGGGGTACAG
AGATCATCAAGACTGCACAGATTCCATGGAAAAATACCAGGCTTCCTTAGAGGC
TGGACTGGAGGAGCCGAGGCAGGAGGCAGGAAGGAGGAGGGAGGAGAGATG
AGGGAGGAGGGTCTGGTGTGTGGGGGCCCTGTACGAACATGCTGCCTTGCCTTG
TTCCAGGGAAAAGGATGTAGCACACAGCCTGCCAGACGGAAGAGGAAGGAAG
GAAGGAAGGAAGGAAGGGGGGGGGGGTGGCACAGAGAGAACTTGTTCCTGTG
GCTTGGGAATGAAGACTGCTGAAAAGGGAGGGGAGCGCTGGGCCCTGGGGAG
AACAACTCAGTATCTCATGGCGACACACCTTGGCTCCAGTCCCACTTCCATGTCC
TAGCCTGTCTCTGTTACTAGCTAGCTCAGTGGTCTGAGGCAGGTCCCCCACCTTC
CAGGGCCGCTATGGAAGCCAAATGAGATTACCTTTGTGAGAAATGAAACTGCAG
CCCTGCCTTAGAGTCTCCATCTTTCCCAGCTTCCCCAGGATCTCCCTGTCTCTCCC
CTCACCTTGATGTTCTCCTTCAGTCTCAGCCCCCAGATCACTGTGCATCATTGTG
CCCTTATGGGGTAGATGGAGACTCCAGTCTCAGCTCTCAGCTCCAGAGACCATC
ATCGCCAGCCCTACTGTGTCCAGAGACTGGCAGTGGGGGTGACCGGAAGTCATT
CAGCCAGCAGACGGCCGGTGGGAGGGCCAGGTGTCCTCCCTCCTGCTGGCGGC
TCATTCTTATGTCAGCTCAGCCTGAGGGTACTAGGCTTTAAGCATCAGTAGGCAG
AGGCTTTCTGAAGTCTGCAGTGCCTAGTCTTGAGGGGGTCCCTCCTTCCTTCCTT
CCTTGTCCCATGCTGACACCAAGAAAGGACTCCAGCCACCTTCAGCTTGGTGGA
GCCTTACCCCTCCCTCCCCACACTGCCACTGAGAGAAGGGTGAAGCCAGATAAT
TAAAAAGGGATGTGGAAAGGCTCTTCTCTTTCCCTCCCTCTGATCTCTGTCAAAG
GACTGCTCTGGACCCCTGGTTTCAACCCATAGCATAGACCTGGAGTTGGACAGG

GTACCTGGGCATCAGCCAGGAGCTAGGACCAGGCAGCAAAAGACATCTGTGCA
AAGTTGGGTAGCAGGCAGCTGTCTAGAGATTTGACGAAAGCACCTGGGCCAGG
GGCTGGGCGGGAGGTCCTGCTGCTCGGCCAGTCAAGGGGCAGAAGCCTATACC
CTTTCTCCTGCGTCCACACAGATGGCAAGTCTGTGGGCAGATGAGAGCAGGAA
GTCCAACCAGGTCCCTGCTGCATCCACCTCTGCTGCCCTGGATTCTGTCTTCATG
AGGAATGGGGTCCCTTAACCCATATCACAGGGTGGGGGATGATCAAGGTACTTG
TGTGTGTGTGTATGTTCACGGGTATGAGCACATGCTTGTGTGTGCCTTTGTGTGC
AGGTACGGGGATGGCCGTGAGCTGTGTCCTTGAAGGAGCTTACTTACCCTTGTT
ACTCACGGATGTTGACTGTTCAGCCTTAGAGAAAACATCTGCTGGGAAGACCAT
CTAGCCGGGGACAGCTGTGCCCCACAGTGGCCATGAAGGGACTTCGAAGATATC
CTATGAAGCACAAGGAGGAGACTTAAGGGACTCTAAAGCCCCCATGCCTCTGGG
AGCCAAGGTGGTGGGGACAGGGACATATGTGATTACTGTCAAGAATTGTGTACT
CACCAGAAGTCTTGGGTTGGGAAGAGGATGAGGAGAACAGTGCTAGAGGTTCC
AGATGTCGGGGCAGGACAGGAGGCTAAGGGACACTTGCTGGTCCCAGACCTCT
CTGACTCCTCTCAGGAGGAGGTTTCCTCCTGAAAAAAGGCTCCTTAGTATGACA
ACACCACAATGTAAATGCTAAGAGGCCAGAGCCCAGGGTATCTGGAATGCTGGG
GACAAAGGGCCAGGACTGACAGGGCTGTGGAACCAAGGTCACCTGGATCTTAG
CGCTCTAAAGATTCACCCAGACCTGCCCCATTGCTGAGGACTTGCGCTTTTCCTC
AGAATTCATCTAGAATCTCTCAGGTAATTTTGCCATGGTTCCTCCCCATGTTCTTT
CCCTGAACAGGCCCAGCCTCCCCACCCCACCACACTGCCCCCATCCTACCCCAC
CCCCGCCCTGATCCTTCCCCAGCCTTCCAGGGGGCTGAAGGTGGCACTATCTAC
CCCTCGCATGGGCAGGATTAGTGGGGCGGGTACTGGAAGAGTGTGGGTTAGAA
ATCCCCCTCTCTGAAGGCAGGGAGCCAGCCAGCCTGGCCTCTTTTCCAGCAGCA
GAATGTAGCCCCAGGCTGCAGTGTGGGGGCGAGAAGGGAGTTGCTGAGGGCTT
CTGGGGCGATAATTGGGCAGCGATTAAATGTTCCCAGAGGAGCTATGGCTGTTGC
CTGTGCCTGTCACCTGTCACTGGGCTCCTGTGTTCGTGGCTTCTGAAGACCCCG
GAGGAGGAAGCAGGGCTTCCTGTGACTTTTCACCCCAGTGGGGCTCAGAGTAG
TGTCTCGGGGTCAGTGGCCTGGATGGACTGCCCTCCATGGAGATAGGAAATGCA
GTGGTCAGAGGGTCAGAGCACTTCAGCCTCGGAAACTTCTCCATTTGTGCCCAA
TCCCCCCTACGGACAGGGGGTGGGGGATCCTGGTCACTGGCCACAGTGCAGCT
GGTTGACACCCTTGCACTCTCCAGCCTGCTCATCCTCAGCTTCCCCACTCTAGGG
GGCTGTCCACCAGCTTCCGTGTGGGTACAGAGAGACTGACTCTGGCCACTGAA
GGGCTACTGCTGGCATGTGTGCAGACAGACCAGGGTCCAGTTGCCCCGTCAACC

CTACCCCATGGCGTAGGACCTTGGTTAACCTTCAGCTCCCTCCAGACTCAGGCCT
GCAGCTCCCGGCTCCCAGGATTCCCCCATAGGAAAACTCTAGTTAGTCCCTATTC
TAGCAATTTCTCTGCTTGTTTCCCCTCCCTGCAGTGATGCCCCTCTGCCTGCCTTC
TGTGTCCGCCTACTCACCTCTAATTAGAAATGCCAGGAGAGCCATTTTAGGGGAG
TTTTTTTTCCCCCAGAAAGACATTCCTCTCAGGGATGGAAGCCCTGGGTGTGTGT
GTGGAGGGGGGGGGGAGGGGGGACAGGTTGGCTCTAAGAGAGGCAGGGCTCC
CCAAAGCTTCTTGAAAACAGAGGAGGGGGCTCTGAGGGATATGAGGTTAGGAG
AGTTCTGTAGGTGTGTGAGCAAAAGTGGGGTGGGAGAAGCTGAGATGTGAGTA
CACGTGGATTACAAGTGGGGGCCACTGGTGTGTGTGTGGAAGGCCATGAGTGG
GGGGCATGAGTCCACAGAGAGGGCCGTGAGTGGGGGGCATGAGTCTACATGGA
AGGGTGTGAGTGGGCTAAGTGCTGGTTGGTATGAGTGTTCAGTGTGTATGTATGA
GTGTGAGAGAGGGGGAGCTGGCAGAGAGGAAAGGGGGTTCTTGCAGGCACCA
TGTGTCTGTGGGCCCCTGTAGGCTTCTGCTGTGGGCAGTGATATGTCCCCAAGTA
ACATCAGGAAATGGGAGACCTAGACCTGTGTTTCTGGCTTGGTGTAAGTCAAGA
AGGATGCCCTCCCCCACTGCACCACAGCGCTGTTGGCTGTTGCCAGGGAATCGG
AAGGGGCCTCCCTCTAGAGGGAATCTCAGCTTCCGGAGCCCCATTCCTTATATGG
CATCTTGCTTTCCTCTGCAGGCTCCTTCCCCTCCTTCCTCCTTCTCCCTCCCTCCT
TCCTCCGGACTAACCTCTTGGAAGGCTTGAGAAGCTTCTGGTCCAGTTGTGGCT
GGGTCCTAGGGACTTCAGGGTTCAGCAATGTTGCCTACACATCTTGATATCCCTA
TGCCCTACCCCAGTGTCTGTACAGGTTCAAGTGATACATCTGTTTCAGGGCACTG
CGGCCCTGTTCAGCCACTGACACACAGACAGGGCCACTCAGTGTCCACAAACC
TATGAAGGGGTCCAGAAAAAGCCTTGCCTGAGTGGAGTTCCCGAACAATATTGG
ACCAGGCTAGGATTTGGACTTATGACTCTTGGGGCAAGGTTGGGCAGGAAGTAG
GTGTGTGTGTGTGTGTGTGTGTGTGTGTGTGTGTGTGTGTGTGTGTGTGTCCCGCTAACCA
AAGGCAGCTGAGGATAGAAGGGTTTGGAGTGAGAGATTCCTACCAGGCACTTCT
GAAGTTGCATACTGTGTGGTCAGGATGGGCAAGAGTCAGGATGGGCGCATCAAA
GATTCAGAGGGGTAACGTCCAGCTGCTCCCTAAGAAAAGAGATCATGAGGCCCC
AGGGCAAACAGTAGGTAATAACCTGCCTTAGCCTCATCCTCGCTGCCCAGGACT
AGGTAGAGGAGAGTGTCTCCGGTCCCCAAAATCCCTCCCGGTGGGAGGCGCGC
AGCAGAGCACATTAGTCACTCGGGGCTGTGAAGGGGCGGGTCCTTGAGGGCAC
CCACGGGAGGGGAGCGAGTAGGCGCGGAAGGCGGGGCCTGCGGCAGGAGAGG
GCGCGGGCGGGCTCTGGCGCGGAGCCTGGGCGCCGCCAATGGGAGCCAGGGCT
CCACGAGCTGCCGCCCACGGGCCCCGCGCAGCATAAATAGCCGCTGGTGGCGGT

TTCGGTGCAGAGCTCAAGCGAGTTCTCCCGCAGCCGCAGTCTCTGGGCCTCTCT
AGCTTCAGCGGCGACGAGCCTGCCACACTCGCTAAGCTCCTCCGGCACCGCAC
ACCTGCCACTGCCGCTGCAGCCGCC
ATCAAGCTTATCGATACCACC
ATGTCTAGACTGGACAAGAGCAAAGTCATAAACTCTGCTCTGGAATTACTCAAT
GAAGTCGGTATCGAAGGCCTGACGACAAGGAAACTCGCTCAAAAGCTGGGAGT
TGAGCAGCCTACCCTGTACTGGCACGTGAAGAACAAGCGGGCCCTGCTCGATGC
CCTGGCAATCGAGATGCTGGACAGGCATCATACCCACTTCTGCCCCCTGGAAGG
CGAGTCATGGCAAGACTTTCTGCGGAACAACGCCAAGTCATTCCGCTGTGCTCT
CCTCTCACATCGCGACGGGGCTAAAGTGCATCTCGGCAACCGCCCAACAGAGA
AACAGTACGAAACCCTGGAAAATCAGCTCGCGTTCCTGTGTCAGCAAGGCTTCT
CCCTGGAGAACGCACTGTACGCTCTGTCCGCCGTGGGCCACTTTACACTGGGCT
GCGTATTGGAGGATCAGGAGCATCAAGTAGCAAAAGAGGAAAGAGAGACACCT
ACCACCGATTCTATGCCCCCACTTCTGAGACAAGCAATTGAGCTGTTCGACCATC
AGGGAGCCGAACCTGCCTTCCTTTTCGGCCTGGAACTAATCATATGTGGCCTGG
AGAAACAGCTAAAGTGCGAAAGCGGCGGGCCGGCCGACGCCCTTGACGATTTT
GACTTAGACATGCTCCCAGCCGATGCCCTTGACGACTTTGACCTTGATATGCTGC
CTGCTGACGCTCTTGACGATTTTGACCTTGACATGCTCCCCGGG
ACTAGTGCCACTAACTTTTCCCTGCTGAAGCAGGCTGGCGACGTGGAGGAAAAT
CCCGGACCGGTCGCCACC
ATGGTGAGCAAGGGCGAGGAGCTGTTCACCGGGGTGGTGCCCATCCTGGTCGA
GCTGGACGGCGACGTAAACGGCCACAAGTTCAGCGTGTCCGGCGAGGGCGAGG
GCGATGCCACCTACGGCAAGCTGACCCTGAAGTTCATCTGCACCACCGGCAAGC
TGCCCGTGCCCTGGCCCACCCTCGTGACCACCCTGACCTACGGCGTGCAGTGCT
TCAGCCGCTACCCCGACCACATGAAGCAGCACGACTTCTTCAAGTCCGCCATGC
CCGAAGGCTACGTCCAGGAGCGCACCATCTTCTTCAAGGACGACGGCAACTAC
AAGACCCGCGCCGAGGTGAAGTTCGAGGGCGACACCCTGGTGAACCGCATCGA
GCTGAAGGGCATCGACTTCAAGGAGGACGGCAACATCCTGGGGCACAAGCTGG
AGTACAACTACAACAGCCACAACGTCTATATCATGGCCGACAAGCAGAAGAACG
GCATCAAGGTGAACTTCAAGATCCGCCACAACATCGAGGACGGCAGCGTGCAG
CTCGCCGACCACTACCAGCAGAACACCCCCATCGGCGACGGCCCCGTGCTGCTG
CCCGACAACCACTACCTGAGCACCCAGTCCGCCCTGAGCAAAGACCCCAACGA
GAAGCGCGATCACATGGTCCTGCTGGAGTTCGTGACCGCCGCCGGGATCACTCT

CGGCATGGACGAGCTGTACAAGTCCGGCCGGA
CTCAGATCTCGAGCTCAAGCTATCCTTACGATGTGCCAGACTATGCC
AGCTTCGAATTCACC
ATGAGCGATCAGCAGCTGGACTGCGCCTTGGACCTGATGAGGCGCCTGCCTCCA
CAGCAGATTGAGAAGAACCTCAGCGATCTGATCGACCTGGTCCCCAGTCTGTGT
GAAGATCTCCTGTCATCTGTTGACCAGCCCCTGAAAATTGCCAGAGACAAGGTG
GTGGGCAAGGATTACCTTTTGTGTGACTACAACAGAGACGGGGACTCCTATAGG
TCACCGTGGAGTAACAAGTATGACCCTCCTTTGGAAGATGGGGCCATGCCATCT
GCTCGGCTCAGAAAGCTGGAGGTAGAGGCCAACAATGCCTTCGACCAATACCG
AGACCTGTATTTTGAAGGTGGGGTCTCATCAGTCTACCTCTGGGATCTTGATCAT
GGCTTTGCTGGAGTGATCCTCATAAAGAAAGCTGGAGATGGATCCAAGAAGATC
AAAGGCTGCTGGGATTCCATCCACGTGGTGGAAGTGCAGGAGAAGTCCAGCGG
CCGTACTGCCCATTACAAGTTGACCTCCACGGTGATGCTATGGCTGCAAACCAAC
AAATCCGGCTCGGGCACCATGAACCTGGGAGGCAGCCTAACCAGACAGATGGA
GAAAGACGAAACTGTGAGTGACTGTTCCCCACACATAGCCAACATCGGGCGCCT
GGTGGAGGACATGGAAAACAAAATCCGAAGCACGCTGAATGAGATCTACTTTG
GAAAAACAAAGGACATCGTCAACGGGCTGAGGTCTGTGCAGACGTTTGCAGAC
AAATCAAAGCAAGAAGCGCTTAAGAACGACCTGGTGGAGGCCTTGAAGAGAAA
GCAGCAGTGTTGACCCGGGATCCACCGGATCTAGATAACTGATCATAATCAGCCA
TACCACATTTGTAGAGGTTTTACTTGCTTTAAAAAACCTCCCACACCTCCCCCTG
AACCTGAAACATAAAATGAATGCAATTGTTGTTGTTAACTTGTTTATTGCAGCTT
ATAATGGTTACAAATAAAGCAATAGCATCACAAATTTCACAAATAAAGCATTTTTT
TCACTGCATTCTAGTTGTGGTTTGTCCAAACTCATCAATGTATCTTAACGCGTCTT
AAG

Transgene with annotation

SalI fragments of transgene were heated then incubated at low temperature to anneal (at the SalI site) before oocyte injection in order to create transgenic mice having high-copy number.

1. linker SalI to AgeI(Bl) 1-18

2. Promotor: mouse Arc SmaI NaeI Fr (7143bp, -7017to +126)

3. tTA seq from pTet-off advanced Tet-Off system Clontech https://www.novoprolabs.com/vector/V11072

4. Synthetic P2A seq

5. EGFP-seq from pEGFP-C1

6. SyntheticOligo for HA tag

7. CapZ beta2 seq TGA=stop

8. pEGFP-C1 SmaI(CCCGGG)-SV40 polyA signal-MluI

9. linker MluI AflII SalI (of a vector, modified pBlueScriptII; lost)

>SmaI (blunted-lost) ligated to AgeI (ACCGG|T blunted-lost) Eco47III SalI of a vector.
>Arc-SARE is underlined (from 241).

GTCGACTAGCGCTACCGG
SalI Eco47III

GGGGGGCCATCTGCTTTCTCCTGGAACCAGAGCTCTAGGGTACAAGTAAACAAA
TACCTGATACAAGATCTTTGTGCAGGTCTGGTTTAAGAGTCACAAAGCCACCAAT
GGCAGGGACAATGTCACCAGTCTCCAGCCAGGGACTCTGGTCCCCACCTCTCCT
TCAGGGCAGGGGCTGAGCAGGTGTAGGGCAGGGTGGGCCCTGGGTGGTGGCAG
GCTCAGCGCACAGAGCCTTCCTGCGTGGGGAAGCTCCTTGCTGCGTCATGGCTC
AGCTATTCTCAGCCTCTCTCCTTTTATGGTGCCGGAAGCAGGCAGGCTGCTGCTG
GGCTGGCTCTGGGAGGTATTTAAATGGAATTGCAGAGAACAGCAGGAAAACAC
AGCCGCCAGGGTGGGGGTGGCAGCCTTGAACGCCACCCTCTAACTGGCATGGG
CATCTGAACTCTCAGCTCTGGGGGTGTCTCATCGGCCAGCTGGCAGTAGCCCCC
CCAACCCCACCCCTGATGGAGAACTGCTCCTGTATGATCCAGCCCAGTGGGCGC
ACAGGGAGTTGGTAGGTGGGCACTCAGACTGGCTAGGGCCCCAGTGGAGCAGA
GATGAGGGGTGGCTGCCAGCCTCCCACCTCAGCCCAGCCTCCCATGGCTCTTAC
TCATTCCTGGTTCGTAACTCTGCCCCTACCCCCAGCTCAGCACTGGCTACCTGGA
TATAGGGAACCCTAATAGTCTTCATTCTTGTCCCTTTGGTAGCCCAAATTCCTGGG
CCATCTCTCCAAAGCAGGGACAGCTCACACATCCAGAACACCAGCCACCAAATC
ATCTCCCCATATCCTGATTCTTTTCTACCCTCTCTACCCTCCATGTCCTCCCCAGGC
TGAGGAGGTCCTGGGTACAATGGCCCTGGCTCAAGGCTGGTGTCCTGGCTAGAA
TAAGGGAGACCTGACTCACCTTGATACCTGGTCTCTGCTACCATCCATGTTCTGC
CCCCACATGGACAAAACCCACATGGCCATGTCCTTGGCCTGGAGTAGCACCCTC
ACACATCTGGCAGTCCCTCTTTATGTCTGGCTGTTGGCTCCTGCATTCCAGTGAT
GGCTGGAAGCAGGTACACTAGGAAAAGCTAAAGGGGGCTACGACTGTGACATA
TATGGTTGCACACAAAACACATTTCCAGACCTGTGCAGATACCAAGATTGACCA
CTCATCTCAGAGACTCTGTGCAGATGCACACAAGATAGACCAAGGCACGCTGTG
CCTGGAGACCCTTCCTAGAGGCACAGAATATGGACAGCTATAGGGAGTCAAGGC
TTTCTCACTTTAACATAGAGTTGGCATTTGTCAACTGAACAGGAGGGTGGACAG
TTGGTCAGAAGGATCCTTGTAGCCACAGGTCCACCCAGTGCTTTCTCAGCCTTC
ATGAAGAAGAAAGAGGAGGAGGAAGAGGAGGAAGAGGAGGAGGAGGAGGAG
GAGGAAGAAGAAGAAGAAGAAGAAGAAGAAGAAGAAGAAGAAGAAGAAGA

AGAAGAAGAAGAAGAAGAAGAAGAAGAAGAAGAAGAAGAAGAAGAAGAAG
ACAAGGAAGAGGAAGAGGAGGAGGAAGGGGAGGAGGAGGAGGAAGAGGAGG
AGGTGCCCTAGTGGCCTTTTAGTACAGTCTGCCTGTTGTATAGTTGAGACCAAGG
TGCTGAATGAGTGCCATGAAGTCACACACAGTCTCAGGGAGATGCAAAAGTTCC
TGTCCACAGCGCTATGGAGGCTTAGGCTAGGCACAGACAGGCTATCTGACATGG
ACCCAAAAGGGGCTTCCTCAGGTCTAGCTCCTGTCTCTTGATTCTACACTTACGC
TGGCCCCCTAGTGTGTGAGTGCTGAGCTCTGTACCCACAATGCCTGCATGGCTTC
GCCTTCCTTGTTCTAGACTATTCTAGCTTGAGCAATGACAATTACATGGACTGCCT
GTTTTCTGCAAGAAGAATCCCCAGGAAGTGCTGGGTGCTCTTTGCCAAGTTCGG
AGGCTCTAAGCAAGGCCACCCACTGGCTGGCGCTGACAAGTTGCAGAAGTAAC
CAAGCTAGAAGCACAACAGAGGGCCATCAGGTGGGGAAAATGAGGCCAGGGA
AGGGGTGGGGACTTAGAGAGACTTACTCATGTTCCCATAATACAAGGGAGTGTC
TTACATAAGGACTCTAGGAGCCTGGAGGTCTCCGTCTGACCTCTAGCCCAATTCC
TAGCCTCTATCATTGTGTGACTTGGATAGGTTTCTGGCCCTCCGTCCCCCTTATGA
ATCATGGCTGGGACCAGGGAGGAACACAGGTCTCTTGAGGACAGACTCCCACA
AACCTGTGTCCTTGTCTACAGTCAGGTGGGCAGTGGGAAATGCCTGGGGTACAG
AGATCATCAAGACTGCACAGATTCCATGGAAAAATACCAGGCTTCCTTAGAGGC
TGGACTGGAGGAGCCGAGGCAGGAGGCAGGAAGGAGGAGGGAGGAGAGATG
AGGGAGGAGGGTCTGGTGTGTGGGGGCCCTGTACGAACATGCTGCCTTGCCTTG
TTCCAGGGAAAAGGATGTAGCACACAGCCTGCCAGACGGAAGAGGAAGGAAG
GAAGGAAGGAAGGAAGGGGGGGGGGGTGGCACAGAGAGAACTTGTTCCTGTG
GCTTGGGAATGAAGACTGCTGAAAAGGGAGGGGAGCGCTGGGCCCTGGGGAG
AACAACTCAGTATCTCATGGCGACACACCTTGGCTCCAGTCCCACTTCCATGTCC
TAGCCTGTCTCTGTTACTAGCTAGCTCAGTGGTCTGAGGCAGGTCCCCCACCTTC
CAGGGCCGCTATGGAAGCCAAATGAGATTACCTTTGTGAGAAATGAAACTGCAG
CCCTGCCTTAGAGTCTCCATCTTTCCCAGCTTCCCCAGGATCTCCCTGTCTCTCCC
CTCACCTTGATGTTCTCCTTCAGTCTCAGCCCCCAGATCACTGTGCATCATTGTG
CCCTTATGGGGTAGATGGAGACTCCAGTCTCAGCTCTCAGCTCCAGAGACCATC
ATCGCCAGCCCTACTGTGTCCAGAGACTGGCAGTGGGGGTGACCGGAAGTCATT
CAGCCAGCAGACGGCCGGTGGGAGGGCCAGGTGTCCTCCCTCCTGCTGGCGGC
TCATTCTTATGTCAGCTCAGCCTGAGGGTACTAGGCTTTAAGCATCAGTAGGCAG
AGGCTTTCTGAAGTCTGCAGTGCCTAGTCTTGAGGGGGTCCCTCCTTCCTTCCTT
CCTTGTCCCATGCTGACACCAAGAAAGGACTCCAGCCACCTTCAGCTTGGTGGA

GCCTTACCCCTCCCTCCCCACACTGCCACTGAGAGAAGGGTGAAGCCAGATAAT
TAAAAAGGGATGTGGAAAGGCTCTTCTCTTTCCCTCCCTCTGATCTCTGTCAAAG
GACTGCTCTGGACCCCTGGTTTCAACCCATAGCATAGACCTGGAGTTGGACAGG
GTACCTGGGCATCAGCCAGGAGCTAGGACCAGGCAGCAAAAGACATCTGTGCA
AAGTTGGGTAGCAGGCAGCTGTCTAGAGATTTGACGAAAGCACCTGGGCCAGG
GGCTGGGCGGGAGGTCCTGCTGCTCGGCCAGTCAAGGGGCAGAAGCCTATACC
CTTTCTCCTGCGTCCACACAGATGGCAAGTCTGTGGGCAGATGAGAGCAGGAA
GTCCAACCAGGTCCCTGCTGCATCCACCTCTGCTGCCCTGGATTCTGTCTTCATG
AGGAATGGGGTCCCTTAACCCATATCACAGGGTGGGGGATGATCAAGGTACTTG
TGTGTGTGTGTATGTTCACGGGTATGAGCACATGCTTGTGTGTGCCTTTGTGTGC
AGGTACGGGGATGGCCGTGAGCTGTGTCCTTGAAGGAGCTTACTTACCCTTGTT
ACTCACGGATGTTGACTGTTCAGCCTTAGAGAAAACATCTGCTGGGAAGACCAT
CTAGCCGGGGACAGCTGTGCCCCACAGTGGCCATGAAGGGACTTCGAAGATATC
CTATGAAGCACAAGGAGGAGACTTAAGGGACTCTAAAGCCCCCATGCCTCTGGG
AGCCAAGGTGGTGGGGACAGGGACATATGTGATTACTGTCAAGAATTGTGTACT
CACCAGAAGTCTTGGGTTGGGAAGAGGATGAGGAGAACAGTGCTAGAGGTTCC
AGATGTCGGGGCAGGACAGGAGGCTAAGGGACACTTGCTGGTCCCAGACCTCT
CTGACTCCTCTCAGGAGGAGGTTTCCTCCTGAAAAAAGGCTCCTTAGTATGACA
ACACCACAATGTAAATGCTAAGAGGCCAGAGCCCAGGGTATCTGGAATGCTGGG
GACAAAGGGCCAGGACTGACAGGGCTGTGGAACCAAGGTCACCTGGATCTTAG
CGCTCTAAAGATTCACCCAGACCTGCCCCATTGCTGAGGACTTGCGCTTTTCCTC
AGAATTCATCTAGAATCTCTCAGGTAATTTTGCCATGGTTCCTCCCCATGTTCTTT
CCCTGAACAGGCCCAGCCTCCCCACCCCACCACACTGCCCCCATCCTACCCCAC
CCCCGCCCTGATCCTTCCCCAGCCTTCCAGGGGGCTGAAGGTGGCACTATCTAC
CCCTCGCATGGGCAGGATTAGTGGGGCGGGTACTGGAAGAGTGTGGGTTAGAA
ATCCCCCTCTCTGAAGGCAGGGAGCCAGCCAGCCTGGCCTCTTTTCCAGCAGCA
GAATGTAGCCCCAGGCTGCAGTGTGGGGGCGAGAAGGGAGTTGCTGAGGGCTT
CTGGGGCGATAATTGGGCAGCGATTAAATGTTCCCAGAGGAGCTATGGCTGTTGC
CTGTGCCTGTCACCTGTCACTGGGCTCCTGTGTTCGTGGCTTCTGAAGACCCCG
GAGGAGGAAGCAGGGCTTCCTGTGACTTTTCACCCCAGTGGGGCTCAGAGTAG
TGTCTCGGGGTCAGTGGCCTGGATGGACTGCCCTCCATGGAGATAGGAAATGCA
GTGGTCAGAGGGTCAGAGCACTTCAGCCTCGGAAACTTCTCCATTTGTGCCCAA
TCCCCCCTACGGACAGGGGGTGGGGGATCCTGGTCACTGGCCACAGTGCAGCT

GGTTGACACCCTTGCACTCTCCAGCCTGCTCATCCTCAGCTTCCCCACTCTAGGG
GGCTGTCCACCAGCTTCCGTGTGGGTACAGAGAGACTGACTCTGGCCACTGAA
GGGCTACTGCTGGCATGTGTGCAGACAGACCAGGGTCCAGTTGCCCCGTCAACC
CTACCCCATGGCGTAGGACCTTGGTTAACCTTCAGCTCCCTCCAGACTCAGGCCT
GCAGCTCCCGGCTCCCAGGATTCCCCCATAGGAAAACTCTAGTTAGTCCCTATTC
TAGCAATTTCTCTGCTTGTTTCCCCTCCCTGCAGTGATGCCCCTCTGCCTGCCTTC
TGTGTCCGCCTACTCACCTCTAATTAGAAATGCCAGGAGAGCCATTTTAGGGGAG
TTTTTTTTCCCCCAGAAAGACATTCCTCTCAGGGATGGAAGCCCTGGGTGTGTGT
GTGGAGGGGGGGGGAGGGGGGACAGGTTGGCTCTAAGAGAGGCAGGGCTCC
CCAAAGCTTCTTGAAAACAGAGGAGGGGGCTCTGAGGGATATGAGGTTAGGAG
AGTTCTGTAGGTGTGTGAGCAAAAGTGGGGTGGGAGAAGCTGAGATGTGAGTA
CACGTGGATTACAAGTGGGGGCCACTGGTGTGTGTGTGGAAGGCCATGAGTGG
GGGGCATGAGTCCACAGAGAGGGCCGTGAGTGGGGGGCATGAGTCTACATGGA
AGGGTGTGAGTGGGCTAAGTGCTGGTTGGTATGAGTGTTCAGTGTGTATGTATGA
GTGTGAGAGAGGGGGAGCTGGCAGAGAGGAAAGGGGGTTCTTGCAGGCACCA
TGTGTCTGTGGGCCCCTGTAGGCTTCTGCTGTGGGCAGTGATATGTCCCCAAGTA
ACATCAGGAAATGGGAGACCTAGACCTGTGTTTCTGGCTTGGTGTAAGTCAAGA
AGGATGCCCTCCCCCACTGCACCACAGCGCTGTTGGCTGTTGCCAGGGAATCGG
AAGGGGCCTCCCTCTAGAGGGAATCTCAGCTTCCGGAGCCCCATTCCTTATATGG
CATCTTGCTTTCCTCTGCAGGCTCCTTCCCCTCCTTCCTCCTTCTCCCTCCCTCCT
TCCTCCGGACTAACCTCTTGGAAGGCTTGAGAAGCTTCTGGTCCAGTTGTGGCT
GGGTCCTAGGGACTTCAGGGTTCAGCAATGTTGCCTACACATCTTGATATCCCTA
TGCCCTACCCCAGTGTCTGTACAGGTTCAAGTGATACATCTGTTTCAGGGCACTG
CGGCCCTGTTCAGCCACTGACACACAGACAGGGCCACTCAGTGTCCACAAACC
TATGAAGGGGTCCAGAAAAAGCCTTGCCTGAGTGGAGTTCCCGAACAATATTGG
ACCAGGCTAGGATTTGGACTTATGACTCTTGGGGCAAGGTTGGGCAGGAAGTAG
GTGTGTGTGTGTGTGTGTGTGTGTGTGTGTGTGTGTGTGTGTGTCCCGCTAACCA
AAGGCAGCTGAGGATAGAAGGGTTTGGAGTGAGAGATTCCTACCAGGCACTTCT
GAAGTTGCATACTGTGTGGTCAGGATGGGCAAGAGTCAGGATGGGCGCATCAAA
GATTCAGAGGGGTAACGTCCAGCTGCTCCCTAAGAAAAGAGATCATGAGGCCCC
AGGGCAAACAGTAGGTAATAACCTGCCTTAGCCTCATCCTCGCTGCCCAGGACT
AGGTAGAGGAGAGTGTCTCCGGTCCCCAAAATCCCTCCCGGTGGGAGGCGCGC
AGCAGAGCACATTAGTCACTCGGGGCTGTGAAGGGGCGGGTCCTTGAGGGCAC

CCACGGGAGGGGAGCGAGTAGGCGCGGAAGGCGGGGCCTGCGGCAGGAGAGG
GCGCGGGCGGGCTCTGGCGCGGAGCCTGGGCGCCGCCAATGGGAGCCAGGGCT
CCACGAGCTGCCGCCCACGGGCCCCGCGCAGCATAAATAGCCGCTGGTGGCGGT
TTCGGT

>+1 ＝GCA of transcript1.https://www.ncbi.nlm.nih.gov/nuccore/NM_018790.3

Arc5’ region of mRNA (GCA to 126GCC, 126bp) does NOT include any known signal like the Dendritic targeting element (An undefined homologous sequence was found in the following underlined CTG to GCC, to Sequence ID: XM_021198619.2 engrailed 100% and others)

GCAGAGCTCAAGCGAGTTCTCCCGCAGCCGCAGTCTCTGGGCCTCTCTAGCTTC
AGCGGCGACGAGCCTGCCACACTCGCTAAGCTCCTCCGGCACCGCACAC
CTGCCACTGCCGCTGCAGCCGCC
ATCAAGCTTATCGATACCACC
HindIII ClaI

> tTA 959-1702 of GenBank: MH325109.1, devoid of stop codon

ATGTCTAGACTGGACAAGAGCAAAGTCATAAACTCTGCTCTGGAATTACTCAAT
XbaI(Southern probe Fr7179-9619 of 9875)
GAAGTCGGTATCGAAGGCCTGACGACAAGGAAACTCGCTCAAAAGCTGGGAGT
TGAGCAGCCTACCCTGTACTGGCACGTGAAGAACAAGCGGGCCCTGCTCGATGC
CCTGGCAATCGAGATGCTGGACAGGCATCATACCCACTTCTGCCCCCTGGAAGG
CGAGTCATGGCAAGACTTTCTGCGGAACAACGCCAAGTCATTCCGCTGTGCTCT
CCTCTCACATCGCGACGGGGCTAAAGTGCATCTCGGCAACCGCCCAACAGAGA
AACAGTACGAAACCCTGGAAAATCAGCTCGCGTTCCTGTGTCAGCAAGGCTTCT
CCCTGGAGAACGCACTGTACGCTCTGTCCGCCGTGGGCCACTTTACACTGGGCT
GCGTATTGGAGGATCAGGAGCATCAAGTAGCAAAAGAGGAAAGAGAGACACCT
ACCACCGATTCTATGCCCCCACTTCTGAGACAAGCAATTGAGCTGTTCGACCATC
AGGGAGCCGAACCTGCCTTCCTTTTCGGCCTGGAACTAATCATATGTGGCCTGG
AGAAACAGCTAAAGTGCGAAAGCGGCGGGCCGGCCGACGCCCTTGACGATTTT
GACTTAGACATGCTCCCAGCCGATGCCCTTGACGACTTTGACCTTGATATGCTGC
CTGCTGACGCTCTTGACGATTTTGACCTTGACATGCTCCCCGGG

> a synthetic seq for P2A self-cleaving peptide seq: TSA-----PVAT

ACTAGTGCCACTAACTTTTCCCTGCTGAAGCAGGCTGGCGACGTGGAGGAAAAT
CCCGGACCGGTCGCCACC

> EGFP reading frame
ATGGTGAGCAAGGGCGAGGAGCTGTTCACCGGGGTGGTGCCCATCCTGGTCGA
GCTGGACGGCGACGTAAACGGCCACAAGTTCAGCGTGTCCGGCGAGGGCGAGG
GCGATGCCACCTACGGCAAGCTGACCCTGAAGTTCATCTGCACCACCGGCAAGC
TGCCCGTGCCCTGGCCCACCCTCGTGACCACCCTGACCTACGGCGTGCAGTGCT
TCAGCCGCTACCCCGACCACATGAAGCAGCACGACTTCTTCAAGTCCGCCATGC
CCGAAGGCTACGTCCAGGAGCGCACCATCTTCTTCAAGGACGACGGCAACTAC
AAGACCCGCGCCGAGGTGAAGTTCGAGGGCGACACCCTGGTGAACCGCATCGA
GCTGAAGGGCATCGACTTCAAGGAGGACGGCAACATCCTGGGGCACAAGCTGG
AGTACAACTACAACAGCCACAACGTCTATATCATGGCCGACAAGCAGAAGAACG
GCATCAAGGTGAACTTCAAGATCCGCCACAACATCGAGGACGGCAGCGTGCAG
CTCGCCGACCACTACCAGCAGAACACCCCCATCGGCGACGGCCCCGTGCTGCTG
CCCGACAACCACTACCTGAGCACCCAGTCCGCCCTGAGCAAAGACCCCAACGA
GAAGCGCGATCACATGGTCCTGCTGGAGTTCGTGACCGCCGCCGGGATCACTCT
CGGCATGGACGAGCTGTACAAGTCCGGCCGGA

> Here is XhoI CTCGA|G
CTCAGATCTCGAGC

> HAtagseq＝(S)YPYDVPDYA
TCAAGCTATCCTTACGATGTGCCAGACTATGCC

>＝SFEFT=Spacer between HA-tag to CapZ
AGCTTCGAATTCACC

>CapZ
ATGAGCGATCAGCAGCTGGACTGCGCCTTGGACCTGATGAGGCGCCTGCCTCCA
CAGCAGATTGAGAAGAACCTCAGCGATCTGATCGACCTGGTCCCCAGTCTGTGT
GAAGATCTCCTGTCATCTGTTGACCAGCCCCTGAAAATTGCCAGAGACAAGGTG
GTGGGCAAGGATTACCTTTTGTGTGACTACAACAGAGACGGGGACTCCTATAGG

TCACCGTGGAGTAACAAGTATGACCCTCCTTTGGAAGATGGGGCCATGCCATCT
GCTCGGCTCAGAAAGCTGGAGGTAGAGGCCAACAATGCCTTCGACCAATACCG
AGACCTGTATTTTGAAGGTGGGGTCTCATCAGTCTACCTCTGGGATCTTGATCAT
GGCTTTGCTGGAGTGATCCTCATAAAGAAAGCTGGAGATGGATCCAAGAAGATC
AAAGGCTGCTGGGATTCCATCCACGTGGTGGAAGTGCAGGAGAAGTCCAGCGG
CCGTACTGCCCATTACAAGTTGACCTCCACGGTGATGCTATGGCTGCAAACCAAC
AAATCCGGCTCGGGCACCATGAACCTGGGAGGCAGCCTAACCAGACAGATGGA
GAAAGACGAAACTGTGAGTGACTGTTCCCCACACATAGCCAACATCGGGCGCCT
GGTGGAGGACATGGAAAACAAAATCCGAAGCACGCTGAATGAGATCTACTTTG
GAAAAACAAAGGACATCGTCAACGGGCTGAGGTCTGTGCAGACGTTTGCAGAC
AAATCAAAGCAAGAAGCGCTTAAGAACGACCTGGTGGAGGCCTTGAAGAGAAA
GCAGCAGTGTTGA

> TGA=StopCodon, then SmaI-pEGFP-C1 vector which includes polyA site
CCCGGGATCCACCGGATCTAGATAACTGATCATAATCAGCCATACCACATTTGTAG
XbaI(Southern probe Fr7179-9619 of 9875)
AGGTTTTACTTGCTTTAAAAAACCTCCCACACCTCCCCCTGAACCTGAAACATAA
AATGAATGCAATTGTTGTTGTTAACTTGTTTATTGCAGCTTATAATGGTTACAAAT
AAAGCAATAGCATCACAAATTTCACAAATAAAGCATTTTTTTCACTGCATTCTAG
TTGTGGTTTGTCCAAACTCATCAATGTATCTTAACGCGTCTTAAGTCGAC
>MluIACGCGT from pEGFP-C1 ligated to short synthetic oligo nucleotide having SalI GTCGAC, which was used to cutout whole transgene from the vector (pBlueScriptII).

Inframe AA seq

tTA　248aminoacids

MSRLDKSKVINSALELLNEVGIEGLTTRKLAQKLGVEQPTLYWHVKNKRALLDAL
AIEMLDRHHTHFCPLEGESWQDFLRNNAKSFRCALLSHRDGAKVHLGTRPTEKQY
ETLENQLAFLCQQGFSLENALYALSAVGHFTLGCVLEDQEHQVAKEERETPTTDSM
PPLLRQAIELFDHQGAEPAFLFGLELIICGLEKQLKCESGGPADALDDFDLDMLPAD
ALDDFDLDMLPADALDDFDLDMLPG

P2Aseq＆Spacer 24aa

TSATNFSLLKQAGDVEENPGPVAT=
ACTAGTGCCACTAACTTTTCCCTGCTGAAGCAGGCTGGCGACGTGGAGGAAAAT
CCCGGACCGGTCGCCACC

EGFP244aa

MVSKGEELFTGVVPILVELDGDVNGHKFSVSGEGEGDATYGKLTLKFICT
TGKLPVPWPTLVTTLTYGVQCFSRYPDHMKQHDFFKSAMPEGYVQERTIF
FKDDGNYKTRAEVKFEGDTLVNRIELKGIDFKEDGNILGHKLEYNYNSHN
VYIMADKQKNGIKVNFKIRHNIEDGSVQLADHYQQNTPIGDGPVLLPDNH
YLSTQSALSKDPNEKRDHMVLLEFVTAAGITLGMDELYKSGRTQ

Spacer　5aa; XhoI then a meaningless alpha-helix spacer in front of HAtag

ISSSS＝ATCTCGAGCTCAAGC

HAtag 9aa

YPYDVPDYA＝TATCCTTACGATGTGCCAGACTATGCC

Spacer　5aa; After HAtag, TTC- is for the meaningless spacer PheGluPhe =FEF while it includes EcoRI）

SFEFT＝AGCTTCGAATTCACC

CapZbeta2　272aa

MSDQQLDCALDLMRRLPPQQIEKNLSDLIDLVPSLCEDLLSSVDQPLKIARDKVVG
KDYLLCDYNRDGDSYRSPWSNKYDPPLEDGAMPSARLRKLEVEANNAFDQYRDL

YFEGGVSSVYLWDLDHGFAGVILIKKAGDGSKKIKGCWDSIHVVEVQEKSSGRTAH
YKLTSTVMLWLQTNKSGSGTMNLGGSLTRQMEKDETVSDCSPHIANIGRLVEDME
NKIRSTLNEIYFGKTKDIVNGLRSVQTFADKSKQEALKNDLVEALKRKQQC*
